# The HSP90 inhibitor HVH-2930 exhibits potent efficacy against trastuzumab-resistant HER2-positive breast cancer

**DOI:** 10.7150/thno.93236

**Published:** 2024-03-31

**Authors:** Minsu Park, Eunsun Jung, Jung Min Park, Soeun Park, Dongmi Ko, Juyeon Seo, Seongjae Kim, Kee Dal Nam, Yong Koo Kang, Lee Farrand, Van-Hai Hoang, Cong-Truong Nguyen, Minh Thanh La, Gibeom Nam, Hyun-Ju Park, Jihyae Ann, Jeewoo Lee, Yoon-Jae Kim, Ji Young Kim, Jae Hong Seo

**Affiliations:** 1 Division of Medical Oncology, Department of Internal Medicine, Korea University College of Medicine, Korea University, Seoul 02841, Republic of Korea.; 2 Brain Korea 21 Program for Biomedical Science, Korea University College of Medicine, Korea University, Seoul 02841, Republic of Korea.; 3 Department of Biomedical Research Center, Korea University Guro Hospital, Korea University, Seoul 08308, Republic of Korea.; 4 Adelaide Medical School, Faculty of Health and Medical Sciences, The University of Adelaide, South Australia 5000, Australia.; 5 Faculty of Pharmacy, PHENIKAA University, Hanoi 12116, Vietnam.; 6 Department of Organic Chemistry, Hanoi University of Pharmacy, Hanoi 10000, Vietnam.; 7 Laboratory of Medicinal Chemistry, College of Pharmacy, Seoul National University, Seoul 08826, Republic of Korea.; 8 School of Pharmacy, Sungkyunkwan University, Suwon, Gyeonggi-do 16419, Republic of Korea.

**Keywords:** C-terminal HSP90 inhibitor, HVH-2930, HER2-positive breast cancer, trastuzumab resistance, cancer stem cells

## Abstract

**Rationale:** Resistance to targeted therapies like trastuzumab remains a critical challenge for HER2-positive breast cancer patients. Despite the progress of several N-terminal HSP90 inhibitors in clinical trials, none have achieved approval for clinical use, primarily due to issues such as induction of the heat shock response (HSR), off-target effects, and unfavorable toxicity profiles. We sought to examine the effects of HVH-2930, a novel C-terminal HSP90 inhibitor, in overcoming trastuzumab resistance.

**Methods:** The effect of HVH-2930 on trastuzumab-sensitive and -resistant cell lines *in vitro* was evaluated in terms of cell viability, expression of HSP90 client proteins, and impact on cancer stem cells. An *in vivo* model with trastuzumab-resistant JIMT-1 cells was used to examine the efficacy and toxicity of HVH-2930.

**Results:** HVH-2930 was rationally designed to fit into the ATP-binding pocket interface cavity of the hHSP90 homodimer in the C-terminal domain of HSP90, stabilizing its open conformation and hindering ATP binding. HVH-2930 induces apoptosis without inducing the HSR but by specifically suppressing the HER2 signaling pathway. This occurs with the downregulation of HER2/p95HER2 and disruption of HER2 family member heterodimerization. Attenuation of cancer stem cell (CSC)-like properties was associated with the downregulation of stemness factors such as ALDH1, CD44, Nanog and Oct4. Furthermore, HVH-2930 administration inhibited angiogenesis and tumor growth in trastuzumab-resistant xenograft mice. A synergistic effect was observed when combining HVH-2930 and paclitaxel in JIMT-1 xenografts.

**Conclusion:** Our findings highlight the potent efficacy of HVH-2930 in overcoming trastuzumab resistance in HER2-positive breast cancer. Further investigation is warranted to fully establish its therapeutic potential.

## Introduction

During times of cellular stress, tumor cells can evolve to rely upon heightened chaperone activity to enhance the stability of oncogenic proteins that drive rapid tumor cell proliferation and survival 1, 2. Over 200 oncogenic HSP90 client proteins have been identified, including transcription factors, tyrosine kinases, and factors related to cancer stemness 3, 4. Many of these are likely to support roles in cell proliferation, DNA repair, and signal transduction. Considering the central role of HSP90 in regulating the activation and dimerization of receptor tyrosine kinases such as HER2, HER3, and EGFR 5, targeting HSP90 represents a promising clinical strategy for treating breast cancer.

Despite 18 documented HSP90 inhibitors entering clinical trials, none have received FDA approval 6. The HSP90 inhibitors in development to date have targeted the ATPase activity of the N-terminal domain, while facing setbacks due to poor bioavailability, lack of clinical efficacy, and dose-limiting toxicities 7. One of the hurdles in developing N-terminal HSP90 inhibitors lies in their tendency to activate heat shock factor 1 (HSF1), triggering the heat shock response (HSR) and subsequent transcription of HSP27, HSP40, HSP70, and HSP90 8, 9. This defensive mechanism paradoxically acts to thwart cell death, posing a substantial challenge 8, 10. In this context, novel strategies that target HSP90 while bypassing the HSR may achieve better therapeutic outcomes.

Amplification or overexpression of the human epidermal growth factor receptor 2 (HER2) gene is present in around 20% of breast cancers, and is associated with greater tumor proliferation, invasion, local disease progression, and distant metastases 11, 12. Since the emergence of trastuzumab as a pioneering HER2-targeted therapy in the late 1990s, there has been notable progress in the development of HER2-targeting monoclonal antibodies, tyrosine kinase inhibitors (TKIs), and advanced HER2-targeting ADCs like T-DM1 and T-DXd 13, 14. Notwithstanding its established efficacy, the existence of both intrinsic and acquired resistance to trastuzumab and TKIs remains a substantial clinical challenge 15. Resistance mechanisms include the presence of truncated p95HER2, activation of pro-survival pathways (involving PTEN depletion, PI3K/AKT activation, and MAPK signaling), and disruption of trastuzumab binding by the mucin 4 and CD44-polymer hyaluronan complex 16, 17.

HER2-positive breast tumors display remarkable heterogeneity, comprising diverse tumor cells including distinct subsets of cancer stem cells (CSCs) 18-20. This particular subset, with dormant properties, evades immune responses, ultimately contributing to metastatic recurrence. Resistance to trastuzumab in particular is associated with mesenchymal stem-like subpopulations displaying the CD44^high^/CD24^low^-phenotype and high ALDH1 activity 21-23. A critical need therefore exists for innovative therapies capable of effectively targeting both cancer stem cells and overcoming trastuzumab resistance to enhance clinical outcomes.

**Figure 1:**
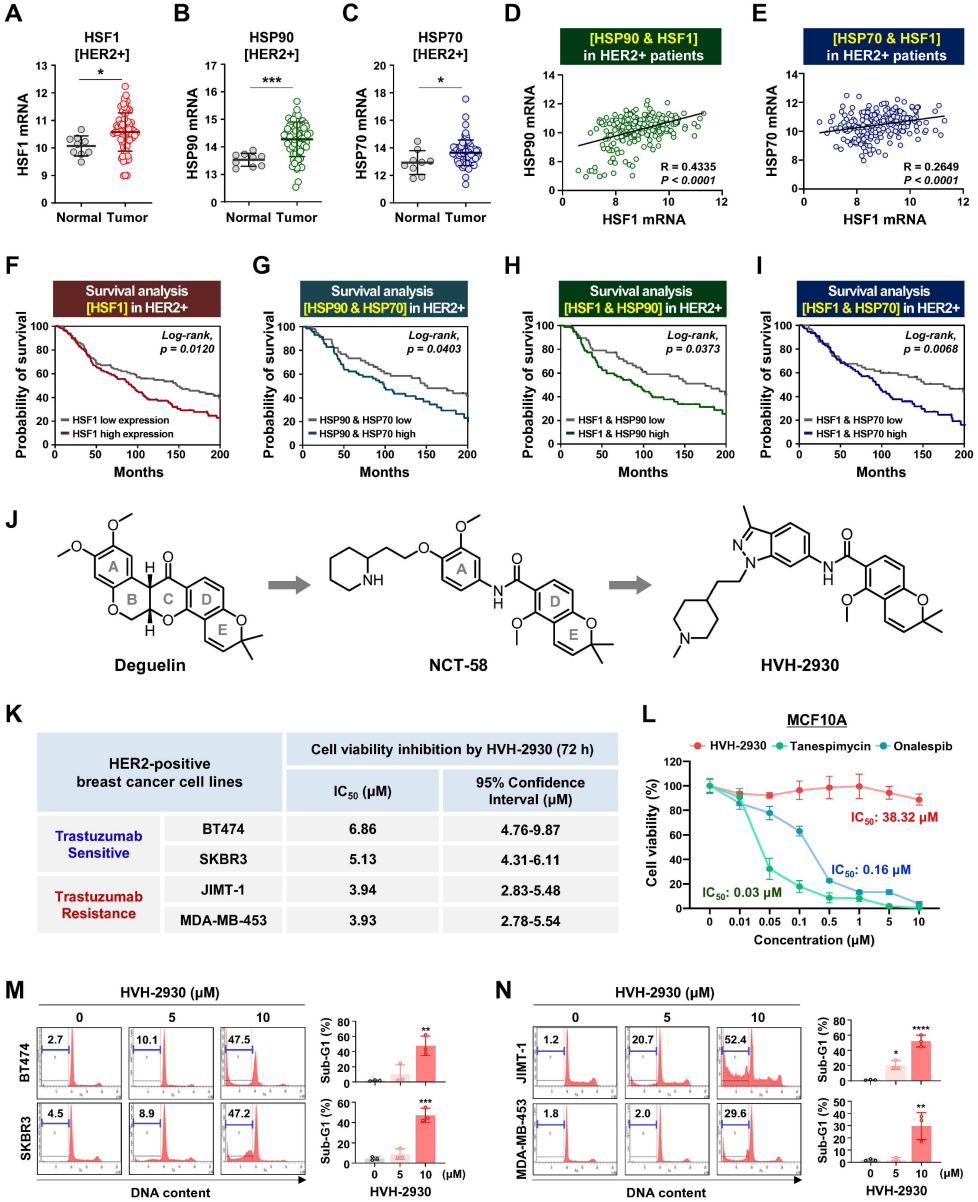
** HVH-2930 reduces cell viability and induces apoptosis in HER2-positive breast cancer cells.** (**A-C**) Comparison of HSF1 mRNA expression between tumor and normal tissue (**A**, *p < 0.05), HSP90 (**B**, ***p < 0.001) and HSP70 (**C**, *p < 0.05) in HER2-positive breast cancer patients derived from the BRCA TCGA dataset. (**D-E**) Correlation of mRNA levels between HSF1 with HSP90 (**D**) and HSP70 (**E**) in HER2-positive breast cancers from the METABRIC cohort. (**F-I**) Kaplan-Meier survival curves show overall survival rates for HER2-positive breast cancer patients with low vs high HSF1 mRNA expression (**F**), HSP90 and HSP70 (**G**), HSF1 and HSP90 (**H**), and HSF1 and HSP70 (**I**). Survival curve comparisons were generated from log-rank (Mantel-Cox) test. (**J**) Chemical structure of deguelin, NCT-58 and HVH-2930. HVH-2930 is a novel C-terminal HSP90 inhibitor optimized by structure-activity relationship studies of NCT-58, a derivative of the B- and C-ring truncated scaffold of deguelin. (**K**) Trastuzumab-sensitive BT474 and SKBR3 and -resistant JIMT-1 and MDA-MB-453 cells were treated with HVH-2930 (0.1-20 μM) or control vehicle (DMSO) for 72 h. Cell viability, 50% inhibitory concentration (IC_50_) and 95% confidence interval (CI_95_) values were determined by MTS assay. (**L**) Normal human mammary gland epithelial MCF10A cells were treated with HVH-2930, tanespimycin and onalespib at concentrations of 0.01-10 µM for 72 h. Cell viability and IC_50_ values were determined by MTS assay. (**M-N**) Sub-G1 populations were quantified by flow cytometry following exposure to HVH-2930 (5-10 μM, 72 h) in trastuzumab-sensitive (**M**, **p < 0.01) and -resistant cell lines (**N**, *p < 0.05). The results are presented as mean ± SEM of at least three independent experiments and analyzed by one-way ANOVA followed by Bonferroni's post hoc test.

On the basis of NCT-58, a derivative of B- and C-ring truncated deguelin reported in our previous study 24, 25, we conducted further investigations into indazole surrogates characterized by isosteric substitution of the dimethoxyphenyl group with substituted indazoles. Through extensive structure-activity relationship (SAR) studies on these indazole surrogates, we have synthesized a novel C-terminal HSP90 inhibitor, HVH-2930, a promising candidate for the treatment of HER2-positive breast cancer (Figure 1).

## Methods

### Reagents and antibodies

The synthesis of HVH-2930 is described in the Supplementary Information. Tanespimycin, onalespib and paclitaxel were purchased from Selleckchem (Houston, TX). Dimethyl sulfoxide (DMSO), propidium iodide (PI), MG132, N-Acetyl cysteine (NAC) and Triton X-100 were purchased from Sigma-Aldrich (St. Louis, MO). Trastuzumab was purchased from Roche Diagnostics Korea Co., Ltd (Seoul, South Korea). Primary antibodies used for immunoblotting and immunostaining were obtained as follows: survivin, HSP90, HSP70, HSF1, HSP27, Cyclin D1 (Santa Cruz, CA); HER2 (CD11), Ki-67, Bcl-2, ALDH1A1, CD44, MDR1 and CD31 (Abcam, MA); AKT, phospho-AKT (S473), mTOR, phospho-mTOR (S2448), Bax, PARP, cleaved-PARP, cleaved-caspase-3, cleaved-caspase-7, cleaved-caspase-8, caspase-9, EGFR, phospho-EGFR (Y1068), HER2, phospho-HER2 (Y1221/1222), HER3, phospho-HER3 (Y1289), MEK1/2, phospho-MEK1/2 (S217/221), ERK1/2, phospho-ERK1/2 (T202/Y204), Oct4 and Nanog (Cell Signaling, CA); p27 (BD bioscience, NJ); phospho-HSF1 (S326) (Bioss, MA); anti-intracellular domain (ICD) HER2 clone 4B5 (Ventana Medical Systems, AZ); and GAPDH (Invitrogen, CA). Secondary antibodies were HRP-conjugated anti-rabbit and mouse IgG (Bio-Rad Laboratories, CA) and Alexa Fluor-488 and -594 goat anti-rabbit IgG (Invitrogen).

### Breast cancer cell culture

The human breast cancer cell lines BT474, SKBR3, MDA-MB-453 (ATCC; American Type Culture Collection, MD), JIMT-1 (DSMZ GmbH, GER) and MDA-MB-231 (PerkinElmer, Inc. CT) were cultured in DMEM, RPMI1640 or MEM (Gibco, MD) containing 10% fetal bovine serum (FBS) and streptomycin-penicillin (100 U/mL). The generation procedure for stable HER2- and p95HER2-overexpressing MDA-MB-231 cells has been described previously 26. The normal human mammary epithelial MCF10A cell line (ATCC) was cultured in MEGM supplemented with hEGF, hydrocortisone, insulin and bovine pituitary extract (SingleQuots^TM^ Kit, Lonza, CA) containing 100 U/mL streptomycin-penicillin. Cells were incubated at 37 °C in an atmosphere of 5% CO_2_. All human cell lines were authenticated by short tandem repeat profiling by Macrogen Inc. (Seoul, South Korea).

### Cell viability assay

Cell viability was determined using the CellTiter 96* Aqueous One Solution Cell Proliferation Assay [MTS, 3-(4,5-dimethylthiazol-2-yl)-5-(3-carboxymethoxyphenyl)-2-(4-sulfophenyl)-2H-tetrazolium] (Promega, Madison, WI) according to the manufacturer's instructions. The formazan product was quantified by measuring the absorbance at 490 nm using a Spectramax Plus 384 microplate analyzer (Molecular Devices, CA).

### Sub-G1 analysis and Annexin V/PI assay

Cells were harvested and fixed with 95% ethanol containing 0.5% Tween-20 for 24 h, incubated with PI (50 µg/mL) and RNase (50 µg/mL) for 30 min. Annexin V/PI assay was performed using a FITC-conjugated Annexin V apoptosis detection kit (BD Biosciences, NJ) according to the manufacturer's protocol. Stained cells were analyzed by flow cytometry using a BD LSRFortessa™ X-20 (BD Biosciences).

### Aldefluor-positivity assay and CD44/CD24 staining

ALDH1 activity was analyzed with an Aldefluor assay kit (Stem Cell Technologies, Canada) according to the manufacturer's instructions. For 45 min (37 ℃), cells were incubated in Aldefluor assay buffer containing ALDH protein substrate BODIPY-aminoacetaldehyde (BAAA, 1 µM / 0.5×10^6^ cells). The ALDH1-specific inhibitor diethylamino-benzaldehyde (DEAB; 50 mM) was defined as the baseline of Aldefluor fluorescence with flow cytometry using a BD Cell Analyzer. For CD44/CD24 analysis, cells (1×10^6^) were immunostained with FITC-conjugated anti-CD24 or PE-conjugated anti-mouse IgG and PE-conjugated anti-CD44 antibodies (BD Biosciences) at 4 °C (30 min) and analyzed by flow cytometry.

### Mammosphere formation assay

BT474 (3.0×10^5^) or JIMT-1 (1.5×10^5^) cells were plated in ultralow attachment dishes and cultured in HuMEC basal serum-free medium (Gibco), supplemented with B27 (1:50, Invitrogen), 20 ng/mL basic fibroblast growth factor (bFGF, Sigma-Aldrich), 20 ng/mL human epidermal growth factor (EGF, Sigma-Aldrich), 4 μg/mL heparin, 1% antibiotic-antimycotic agent, and 15 μg/mL gentamycin. The numbers and volumes of mammospheres were determined under an Olympus CKX53 inverted microscope (Olympus Life Science). Mammosphere volumes were calculated by the formula: volume = 4/3*3.14(π)*r3 (r: radius).

### Immunoblot analysis

Cells were lysed in a solubilizing buffer (30 mM NaCl, 0.5% Triton X-100, 50 mM Tris-HCl; pH 7.4) containing a cocktail of phosphatase and protease inhibitors (cOmplete™, Roche), and incubated on ice for 45 min. Cell lysates were collected (14,000 rpm, 4 °C, 20 min) and protein concentrations were measured with a Bradford protein assay kit (Bio-Rad). Equal quantities of protein (30 μg) were subjected to SDS-PAGE and electrotransferred onto a polyvinylidene fluoride (PVDF) membrane (Millipore, MO). The membranes were incubated overnight at 4 °C with primary antibodies diluted in 5% BSA [PARP (1:2000), cleaved PARP (1:2000), cleaved caspase-3 (1:1000), cleaved caspase-7 (1:1000), cleaved caspase-8 (1:1000), cyclin D1 (1:2000), survivin (1:2000), caspase-9 (1:2000), Bcl-2 (1:1000), Bax (1:1000), HER2 (1:2000), phospho-HER2 (1:1000), HER3 (1:2000), phospho-HER3 (1:1000), EGFR (1:2000), phospho-EGFR (1:2000), AKT (1:2000), phospho-AKT (1:1000), MEK 1/2 (1:2000), phospho-MEK 1/2 (1:1000), ERK 1/2 (1:2000), phospho-ERK 1/2 (1:1000), Oct4 (1:500), Nanog (1:500), HSP90 (1:5000), HSP70 (1:5000), HSF1 (1:3000), phospho-HSF1 (1:1000), MDR1 (1:1000) and GAPDH (1:10000)], followed by incubation with HRP-conjugated anti-rabbit and mouse IgGs (1:1000-1:5000). Signal intensity was detected using an Enhanced Chemiluminescence Kit (Thermo Fisher Scientific, IL) on X-ray film (Agfa Healthcare, Belgium) and quantitated using AlphaEaseFC software (Alpha Innotech, CA).

### Measurement of reactive oxygen species (ROS) generation

2', 7'-Dichlorodihydrofluorescin diacetate (DCFH-DA; Cell Biolabs Inc. CA) was used to evaluate ROS generation. Cells were treated with HVH-2930 (0 and 10 μM) for 1-12 h and then stained with 1 mM DCFH-DA for 30 min at 37 °C. The uptake of DCF fluorescence (excitation 480 nm and emission 530 nm) was measured by flow cytometry.

### Intracellular ATP assay

BT474 (3.0×10^4^), SKBR3 (1.5×10^4^), JIMT-1 (1.0×10^4^) and MDA-MB-453 cells (3.0×10^4^) were seeded in 96-well white/clear flat bottom plates (Corning, NY). After incubation for 24 h, the cells were treated with HVH-2930 (0, 5 and 10 μM) for 48 h and then incubated with CellTiter-Glo® ATP assay luminescent reagent (Promega, WI) at 37 °C for 10 min. The ATP contents were determined by measuring luminescence using a Varioskan LUX™ multimode microplate reader (Thermo Fisher Scientific).

### Immunocytochemistry

Cells on 8-well chamber slides (BD Biosciences) were fixed with 4% paraformaldehyde, washed with PBS, and incubated with 0.2% Triton X-100 for 10 min. The cells were incubated with primary antibodies in antibody diluent (Dako, Denmark) overnight at 4 °C, and then incubated with Alexa Fluor-488 or -594 conjugated secondary antibodies (Invitrogen). Cells were mounted with ProLong Gold Antifade Reagent with DAPI (Thermo Fisher Scientific). Images were acquired using a confocal microscope (Carl Zeiss, GER), and analyzed using the intensity profile tool.

### Immunoprecipitation

A Dynabeads™ Protein G Immunoprecipitation Kit (Invitrogen) was used to evaluate protein interactions according to the manufacturer's protocol. Cells were lysed in Pierce^TM^ IP lysis buffer (Thermo Fisher Scientific) containing a cocktail of phosphatase and protease inhibitors. The supernatant was collected after centrifugation (15,000 rpm, 4 °C, 20 min). Equal quantities (1000 µg) were incubated with 10 µg of rabbit polyclonal anti-HER2 antibody conjugated to Dynabeads at 4 °C overnight. The protein complexes were recovered by boiling the beads in a mixture of SDS-PAGE sample buffer and elution buffer (1:1), followed by SDS-PAGE and immunoblotting.

### Animals and *in vivo* xenograft experiment

All animal procedures were carried out in accordance with animal care guidelines approved by the Korea University Institutional Animal Care and Use Committee (IACUC: KOREA-2021-0058). Five-week-old female BALB/c nude mice (NARA Biotech Animal Center, South Korea) were housed in a specific pathogen-free environment and acclimated for 1 week prior to the study with free access to food and water. JIMT-1 cells (3.0×10^6^) were inoculated into the fourth mammary fat pads of 6-week-old female BALB/c nude mice (n = 6/each group). When the average tumor volume reached 100 mm^3^, the animals were randomized into 2 groups, receiving intraperitoneal administration of either vehicle (DMSO/saline, 1:9) or HVH-2930 (20 mg/kg) every other day for 40 days. For the combination study, the animals were randomized into 4 groups (n = 6/each group) and received intraperitoneal administrations of vehicle (DMSO/saline, 1:9), HVH-2930 (10 mg/kg, every other day), paclitaxel (4 mg/kg, once a week), or a combination of HVH-2930 and paclitaxel for 40 days. Tumor volumes and body weight were measured twice weekly after the initial treatment and calculated using the formula V = (Length×Width2)/2. For a xenograft model of experimental metastasis, HER2- and p95HER2-overexpressing MDA-MB-231 cells (1×10^6^) were injected into the tail veins of 6-week-old female NOD/SCID mice, following exposure to control solvent or HVH-2930 (10 μM) for 24 h *in vitro*. After 45 days, the mice were then anesthetized and assessed using a NightOWL II LB 983 *In vivo* BLI System (Berthold Technologies, TN). For *in vivo* imaging, a chemiluminescent luciferase substrate, D-luciferin sodium salt (Abcam) was administered intraperitoneally at a dose of 150 mg/kg in 100 µL PBS prior to imaging. The captured images were quantified using IndiGo™. For the study of trastuzumab responsiveness, the animals were randomly divided into two groups (n = 7/each group) and received intraperitoneal administrations of vehicle (0.9% of NaCl in water) or trastuzumab. The trastuzumab treatment was initiated with a loading dose of 4 mg/kg, followed by a maintenance dose of 2 mg/kg. Tumor volume was measured every 5 days for 40 days.

### Serum biochemical analysis for liver and renal injury biomarkers

After sacrifice, blood samples were collected from each animal, and serum activities of alanine aminotransferase (ALT), aspartate aminotransferase (AST) and blood urea nitrogen (BUN) levels were assessed with an assay kit following the manufacturer's protocol (Sigma-Aldrich). All assays were measured with a Spectra Max 190 (Molecular Devices) and analyzed using SoftMax Pro 7 software.

### Immunohistochemistry (IHC) and in-situ localization of apoptosis (TUNEL)

After removal, tumors were fixed in 10% neutral-buffered formalin before paraffin embedding. Tissue sections (5 µm) were mounted on positively-charged microscope slides and then deparaffinized with xylene and dehydrated through a series of graded alcohol solutions. Antigen retrieval was performed by boiling the tissue sections in citrate buffer (pH 6.0). Tissue sections with primary antibodies [CD31; 1:200, Ki-67; 1:200, HER2; 1:100, ICD HER2 clone 4B5, CD44; 1:200, ALDH1A1; 1:100, HSP70; 1:200, HSF1; 1:200 or HSP90; 1:200] in antibody-diluent were incubated overnight at 4 °C. For secondary antibody reactions, the sections were incubated with Alexa Fluor® -488 or -594 conjugated secondary antibodies at room temperature for 2 h and mounted with DAPI. *In situ* TUNEL assays were carried out on tissue sections using an *In situ* Cell Death Detection Kit (Roche Applied Sciences, GER) in accordance with the manufacturer's instructions. For histopathological analysis, selected organ tissues (lung, liver, kidney and tumor) were stained with hematoxylin and eosin (H&E). Images were taken using a slide scanner (Axio Scan.Z1, Zeiss).

### C-terminal HSP90 inhibition assay

An HSP90α (C-Terminal) Inhibitor Screening Assay Kit (BPS Bioscience, CA) was used to evaluate inhibition of the interaction between C-terminal HSP90α and its co-chaperone peptidylprolyl isomerase D (PPID) by HSP90 inhibitors, as previously described 24, 27. The HSP90 inhibitors (HVH-2930, novobiocin, tanespimycin, and onalespib) were added at 500 µM to the reaction buffer containing both diluted HSP90α (1.5 ng/µl) and PPID (10 ng/µl) in an Optiplate-384 (PerkinElmer, Inc. USA), and then reacted with Detection AlphaLISA® Acceptor Beads and Streptavidin-conjugated donor beads (PerkinElmer, Inc. USA). C-terminal HSP90α:PPID binding activity was analyzed using an AlphaScreen® microplate reader (Varioskan LUX™, Thermo Fisher Scientific, Rockford, IL).

### N-terminal HSP90 binding activity assay

An HSP90α N-terminal domain Assay Kit (BPS Bioscience, CA) was used according to the manufacturer's protocol. This competitive binding assay assesses the binding of fluorescently labeled geldanamycin, an N-terminal HSP90 inhibitor, to purified recombinant HSP90α. Briefly, HVH-2930, novobiocin, tanespimycin, or onalespib (0-1000 nM) dissolved in DMSO was incubated with the reaction mixture containing both FITC-labeled geldanamycin (100 nM) and HSP90α (17 ng/µl) for 3 h at room temperature. The N-terminal HSP90 binding activity was determined by fluorometric detection (λ_ex_ 485 nm, λ_em_ 530 nm) using a microplate reader (SpectraMax Gemini EM, Molecular Devices).

### Wound healing assay

To analyze kinetic migration, JIMT-1 cells were grown to ~90% confluency in 96-well plates (Essen ImageLock, MI). Physical wounds were created using a 96-pin Wound Maker device before washing in media to prevent reattachment of removed cells. Cells were treated with HVH-2930 (0-20 µM) after wound creation. The wound fields were monitored and images were captured hourly for up to 72 h using an IncuCyte™ ZOOM® live-cell Imaging System (Essen BioScience). Wound confluency was assessed using the IncuCyte™ Scratch Wound Analysis Software Module.

### Surface Plasmon Resonance (SPR) analysis

SPR analyses were performed at 25 ºC using Biacore 3000 optical biosensors equipped with HC1000M sensor chips (Biacore AB). An activated HC1000M chip was immobilized with recombinant human HSP90AA1 protein at a concentration of 66.5 µg/ml in 5 mM acetate buffer at pH 4, followed by blocking with 1 M ethanolamine. HSP90 inhibitors were initially dissolved in 100% DMSO and then further diluted with the running buffer (comprising 10 mM phosphate, 137 mM NaCl, 2.7 mM KCl, 0.005% Tween20, 1% DMSO, pH 7.4) to achieve final concentrations of 40, 20, 10, 5, 2.5, 1.25, 0.625, and 0 µM. After dilution, HSP90 inhibitors were injected over the HSP90AA1-immobilized sensor chip at a flow rate of 30 µl/min. Each response curve was generated by subtracting the background signal simultaneously obtained from the control flow cell, and the binding data was analyzed using BIAevaluation Software.

### Molecular modeling and docking analysis

Computational docking of HVH-2930 onto the C-terminal domain of hHSP90α homology model was conducted as described previously 28, 29. The docking was performed using Tripos Sybyl-X 2.1 in the Windows 7 operating system. All ligands were prepared in mol2 format using the sketch module and minimized. Tripos force field and Gasteiger-Hückel charge; conjugate-gradient method with convergence criterion of 0.001 kcal mol-1.Å-1 and max iteration to 10000 for energy minimization were used. Our previously reported homology model of hHSP90:ATP complex was used as a receptor for docking 29. The protomol was defined with twenty amino acid residues adjacent to the ATP binding pocket and the binding cavity of the hHSP90 homodimer. The threshold parameter was set to 0.50 with a bloat parameter of 5 Å. Docking was conducted using the default settings of Surflex-Dock GeomX, generating 50 maximum poses per ligand and performing CScore (consensus score) calculations 30. The binding conformation of HVH-2930 was selected by considering the Surflex-Dock score, CScore, and visual inspection. Visualization and rendering of docking models were performed on a Maestro graphic user interface in Schrödinger 2020-1 and Benchware 3D explorer program.

### Public dataset source and bioinformatics analysis

Gene expression in normal and tumor tissues was analyzed using the publicly available METABRIC and TCGA-BRCA datasets in cBioPortal (http://www.cbioportal.org). RNA-Seq dataset analysis using the Gene Expression Omnibus (GEO) dataset GSE161420 31 from a patient-derived xenograft (PDX), derived from a HER2-positive patient with trastuzumab resistance, in the presence or absence of trastuzumab (6 mg/kg, once a week for 3 weeks). mRNA expression levels were quantified using the Reads Per Kilobase of Exon Per Million Mapped Reads (RPKM) metric. For correlation analysis of several genes, Pearson's correlation coefficients (R) were calculated using the TCGA cohort. Overall survival data were acquired by Kaplan-Meier analysis using the METABRIC cohort. Significance was determined through the log-rank test at *p* < 0.05.

### Statistical analysis

All data were analyzed using GraphPad Prism 9.0 statistical software (San Diego, CA). The results are presented as mean ± SD of at least three independent experiments. Data were analyzed by Student's t-test and one- or two-way ANOVA, as appropriate. Significance between multiple groups was determined using the Bonferroni post hoc test and defined at *p* < 0.05.

## Results

### Prognostic significance of heat shock proteins (HSPs) in patients with HER2-positive breast cancer

The HSP90 chaperone machinery is widely recognized for its direct regulatory role in stabilization and activation of the HER2 receptor 32. In a previous study, it has been demonstrated that HER2 overexpression triggers activation of the HSF1-HSP90 axis and subsequently leads to the stabilization of HSP90 client proteins such as AKT, mTOR and HSF1 itself, thereby promoting tumor growth in HER2-positive breast cancer 33. Initially, we analyzed cohort studies exploring the association of mRNA expression between the HSP90 chaperone complex and HER2. In the publicly-available dataset for HER2-positive breast cancer patients, mRNA expression levels of HSF1, HSP90 (HSP90AA1) and HSP70 (HSPA1A) are simultaneously upregulated in tumor tissues relative (*p* < 0.05, Figure 1A-C), with a substantial positive correlation between HSF1 and either HSP90 (*p* < 0.0001, Figure 1D) or HSP70 (*p* < 0.0001, Figure 1E). HER2-positive breast cancer patients with high HSF1 mRNA expression showed a notably lower probability of overall survival (Log-rank, *p* = 0.012, Figure 1F), and concurrent overexpression between HSP90 and HSP70 (*p* = 0.0403, Figure 1G), HSF1 and HSP70 (*p* = 0.0373, Figure 1H), and HSF1 and HSP70 (*p* = 0.0068, Figure 1I) were also associated with relatively worse overall survival. Additionally, analysis of public GEO datasets revealed a significant increase in the mRNA expression of HSP90 and HSF1 in patient-derived xenograft (PDX), originating from a HER2-positive patient exhibiting resistance to trastuzumab (Figure S1).

### HVH-2930 induces apoptosis in HER2-positive breast cancer cells

HVH-2930 is a novel C-terminal HSP90 inhibitor generated through structure-activity relationship (SAR) studies on indazole surrogates based on NCT-58, a derivative of deguelin. HVH-2930 is structurally distinct from NCT-58 and other known C-terminal inhibitors due to its inclusion of a 3-methylindazole ring, replacing the 3,4-dimethoxyphenyl group as the A-ring. Although both the 3-methylindazole and 3,4-dimethoxyphenyl groups engage in similar π-π interactions with the ammonium ion of Lys615 in one of the HSP90 chains, the 3-methylindazole ring offers enhanced pharmacokinetic benefits over the metabolically less stable 3,4-dimethoxyphenyl group (Figure 1J, Supplementary information 1).

We first sought to evaluate the effects of HVH-2930 on cell viability and apoptosis in HER2-positive breast cancer cells. Cell viability was dose-dependently reduced in both trastuzumab-sensitive [BT474 and SKBR3] and -resistant cells [JIMT-1 and MDA-MB453 34-36 following exposure to HVH-2930 (0.1-20 μM, 72 h) (Figure S2). The IC_50_ values of HVH-2930 were calculated to be 6.86 μM, 5.13 μM, 3.94 and 3.93 μM in BT474, SKBR3, JIMT-1 and MDA-MB-453 cells, respectively (Figure 1K). MCF10A cells were less sensitive to HVH-2930 treatment (IC_50_: 38.32 μM), while the first-generation N-terminal HSP90 inhibitor tanespimycin and second-generation onalespib exhibited significant cytotoxicity even in non-malignant cells, with IC_50_ values of 0.03 and 0.16 μM, respectively (Figure 1L). HVH-2930 treatment (5-10 μM, 72 h) effectively induced apoptosis in both trastuzumab-sensitive and -resistant cells, as evidenced by a marked accumulation in the sub-G1 population (*p* < 0.01, Figure 1M) and a significant increase in the number of early and late apoptotic cells (*p* < 0.05, Figure 1N).

### HVH-2930-induced apoptosis is associated with mitochondrial dysfunction

**Figure 2:**
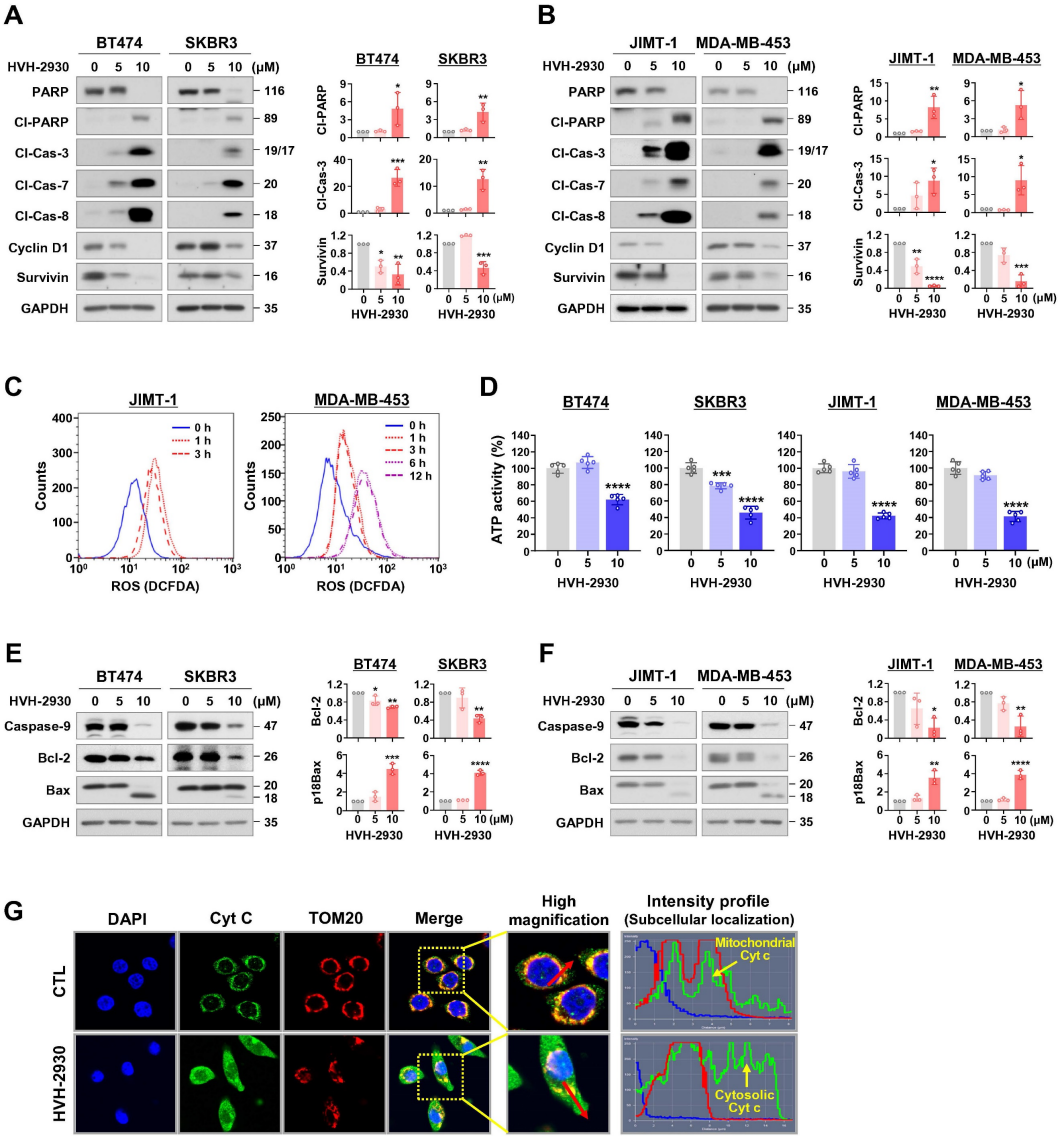
** HVH-2930-induced apoptosis is accompanied by activation of effector caspases and mitochondrial dysfunction.** (**A-B**) Effect of HVH-2930 on the expression of apoptosis-related proteins. Immunoblot analyses for PARP, cleaved-PARP, cleaved caspase-3, cleaved caspase-7, cleaved caspase-8, cyclin D1, and survivin in trastuzumab-sensitive (**A**) and -resistant cells (**B**) after treatment with HVH-2930 (5-10 μM, 72 h). GAPDH was used as an internal control. Quantitative graphs of protein content are shown in the right panels, (*p < 0.05). (**C**) JIMT-1 and MDA-MB-453 cells were treated with HVH-2930 (10 μM, 1-12 h), and intracellular ROS generation was determined by DCF-DA staining using flow cytometry. Data was analyzed using the FlowJo v10.1 software. (**D**) Intracellular ATP levels in BT474, SKBR3, JIMT-1 and MDA-MB-453 treated with HVH-2930 (5-10 μM, 48 h) were measured using a luciferase-based ATP assay (***p < 0.001). (**E-F**) Effect of HVH-2930 (5-10 μM, 72 h) on the expression of caspase-9, Bcl-2, and Bax in trastuzumab-sensitive (**E**) and -resistant cells (**F**). Ratio of Bcl-2/GAPDH and truncated p18Bax/GAPDH (right panels, *p < 0.05). (**G**) Release of cytochrome c from mitochondria to the cytosol following HVH-2930 treatment. JIMT-1 cells were treated with HVH-2930 at 10 μM for 24 h and immunostained for cytochrome c (green) and TOM20 (red, mitochondria) with DAPI (blue, nucleus). Signal intensity of cytochrome c (green line) and cellular localization (yellow arrows) were determined by confocal microscopy using the intensity profile tool. Cyt c, cytochrome c; TOM20, translocase of outer mitochondrial membrane 20.

Several studies have shown that HSP90 inhibitors disrupt mitochondrial integrity in various cancer cells, including neuroblastoma, cervical cancer, glial and prostate cancer cells, leading to apoptosis accompanied by the loss of mitochondrial membrane potential and the generation of oxidative stress 37, 38. We next sought to determine whether the early events of mitochondrial dysfunction are associated with HVH-2930-induced apoptosis. HVH-2930 treatment (5-10 μM, 72 h) resulted in the activation of caspase-3, -7 and -8 and PARP cleavage, leading to downregulation of the pro-survival factors cyclin D1 and survivin in both trastuzumab-sensitive and -resistant cells (*p* < 0.05, Figure 2A-B and Figure S3). This phenomenon coincided with the induction of excessive reactive oxygen species (ROS) accumulation at an early stage (1-6 h) and depletion of ATP synthesis during apoptotic cell death (*p* < 0.001, Figure 2C-D and Figure S4). Following exposure to HVH-2930, the alterations in mitochondrial proteins were observed, as evidenced by the downregulation of Bcl-2 and the increased Bax cleavage, as a potent proapoptotic 18 kDa fragment, as well as a decrease of procaspase-9 (*p* < 0.05, Figure 2E-F). To identify the induction of cytochrome c release as a consequence of these events, we conducted double-immunocytochemistry for cytochrome c and the translocase of outer membrane 20 (TOM20) as a mitochondrial marker. The fluorescence intensity profile revealed that cytochrome c was markedly localized from the mitochondria into the cytosol following HVH-2930 treatment (10 μM) at 24 h (Figure 2G and Figure S5). Our findings suggest that HVH-2930-induced apoptosis is triggered by intracellular ROS accumulation and subsequently deregulating the mitochondrial Bcl-2 and Bax proteins, leading to the release of cytochrome c and activation of effector caspases. We further examined the effect of trastuzumab on the apoptosis pathway including ROS production, and expression of mitochondrial proteins in trastuzumab-resistant cells. Trastuzumab did not affect ROS production, the protein levels of Bcl-2 and Bax, or the expression of effector caspases such as caspase-9 and cleaved caspase-3 (Figure S6).

### HVH-2930 targets the C-terminal HSP90 without inducing the HSR

**Figure 3:**
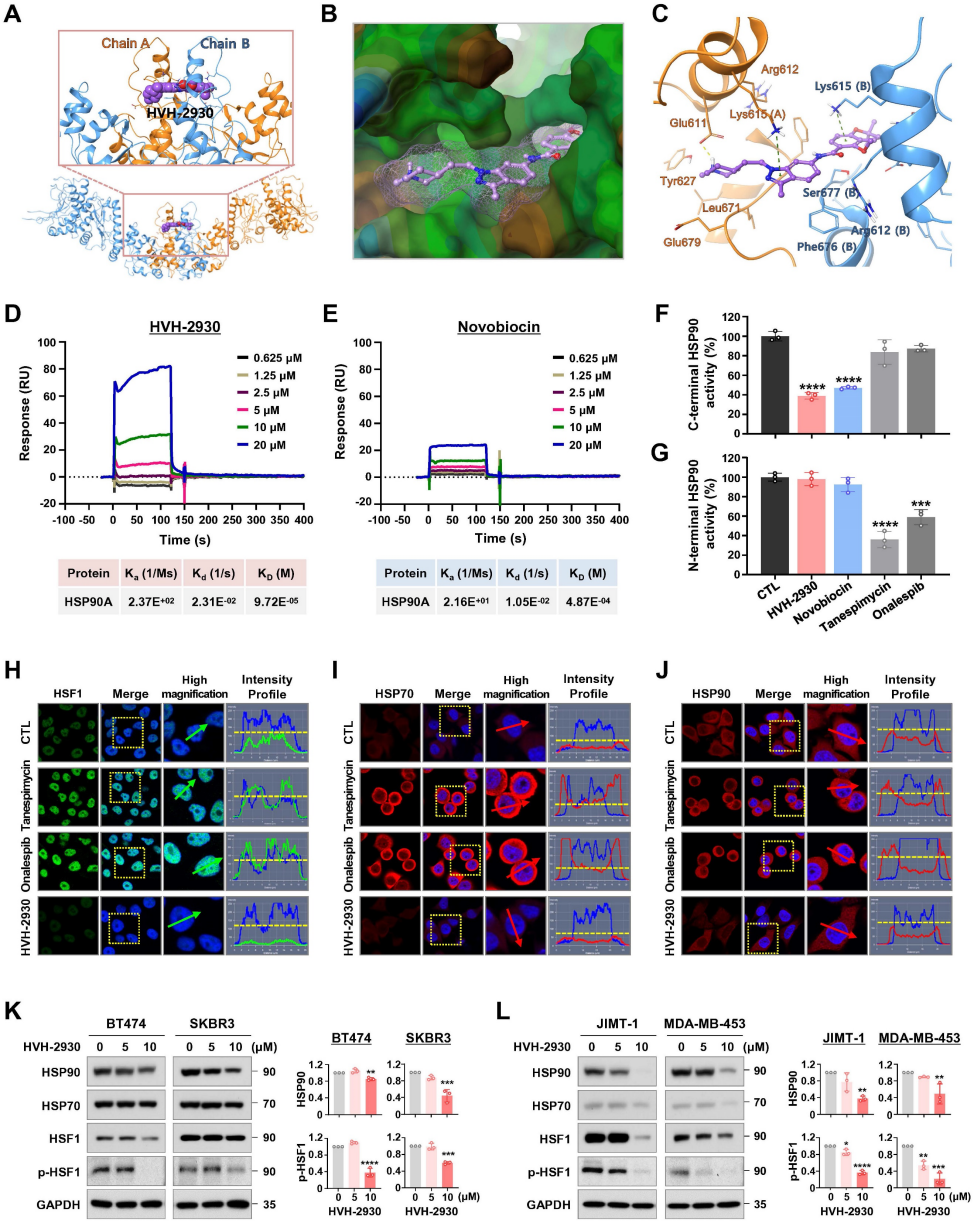
** HVH-2930 targets the C-terminal domain of HSP90 without triggering the HSR.** (**A-C**) Molecular docking model of HVH-2930 with the C-terminal domain of hHSP90α. (**A**) Binding pose of HVH-2930 in the dimerization interface. HVH-2930 is shown in a space-filling model. (**B**) Lipophilicity property surface map (brown color: hydrophobic, blue color: hydrophilic) in the binding cavity. The Connolly surface of HVH-2930 is shown as mesh. (**C**) Binding pose of HVH-2930 in the open state conformation of hHSP90 (Surflex-Dock score = 7.264). Chain A of hHSP90 is rendered in orange ribbon, and chain B is blue ribbon. HVH-2930 is displayed as a ball-and-stick model. Hydrogen bonds and π-cation interactions are represented as yellow and green dashed lines, respectively. (**D-E**) Surface plasmon resonance (SPR) binding curve of (**D**) HVH-2930- and (**E**) novobiocin-HSP90 interaction. The indicated concentrations of HVH-2930 and novobiocin (0.625-20 µM) were passed over immobilized human HSP90AA1 protein on HC1000M sensor chips. The kinetic interaction of HVH-2930 (K_D_ = 97.2 µM) and novobiocin (K_D_ = 487 µM) with HSP90a was determined with SPR analyses (n = 3). (**F-G**) Effect of HVH-2930 on inhibition of the C-terminal or N-terminal domain of HSP90. (**F**) The inhibitory effect of HSP90 inhibitors (HVH-2930, novobiocin, tanespimycin or onalespib at 500 μM) on HSP90α (C-terminal):PPID binding activity was determined using an HSP90α (C-terminal) inhibitor screening assay (***p < 0.001). (**G**) The competitive HSP90α (N-terminal) binding activity of HSP90 inhibitors (1000 nM) with FITC-labeled geldanamycin was determined with an HSP90α N-terminal domain assay (***p < 0.001). The results are presented as mean ± SD of at least three independent experiments analyzed by unpaired Student's t-test. (**H-J**) Comparison of the effects of HVH-2930, tanespimycin and onalespib on induction of HSR. SKBR3 cells were immunostained for HSF1 (green, **H**), HSP70 (red, **I**) or HSP90 (red, **J**) with DAPI (nuclei, blue), following exposure to HVH-2930, tanespimycin or onalespib (300 nM) for 24 h. The fluorescence intensity of these proteins is represented in arbitrary units as defined by the software (yellow dotted line). (**K-L**) Immunoblot analyses of HSP90, HSP70, HSF1 and phospho-HSF1 (S326) protein expression in trastuzumab-sensitive (**K**) and -resistant cells (**L**) following exposure to HVH-2930 (0-10 μM, 72 h). Quantitative graphs represent the ratio of protein content in the presence or absence of HVH-2930 (**p < 0.01).

To elucidate the molecular mechanism underlying the interaction between HVH-2930 and the C-terminal domain of hHSP90α, we performed computational docking on a homology model of the open conformation of the protein. HVH-2930 fits into the predicted ATP-binding pocket 28 and cavity at the interface of the hHSP90 homodimer, similar to a latch (Figure 3A-B). This model suggests that the drug stabilizes the open conformation of the C-terminal domain of the hHSP90α homodimer. Two aromatic ring moieties, indazole and chromene, are almost coplanar and involved in π-cation interactions with the Lys615 of each hHSP90 chain. The protonated 1-methylpiperidine group localizes to the region which was predicted to be a ribose binding site in the ATP-binding pocket. The Glu611 in chain A may form a strong H-bond with protonated 1-methylpiperidine (Figure 3C). The resulting model suggests that HVH-2930 is a potent C-terminal hHSP90 inhibitor because it not only inhibits the binding of ATP but also stabilizes its open conformation.

The interaction between HVH-2930 and HSP90α was confirmed using surface plasmon resonance (SPR) analysis. The SPR data revealed that HVH-2930 exhibited a dose-dependent increase in binding to HSP90α and exhibited a higher binding affinity than novobiocin. The equilibrium dissociation constant (K_D_) value for the binding of HVH-2930 to HSP90α was determined to be 97.2 μM (Figure 3D), while novobiocin showed a relatively lower affinity at 487 μM (Figure 3E).

The C-terminal domain of HSP90 comprises a highly conserved pentapeptide sequence (MEEVD), which interacts with peptidyl-prolyl cis-trans isomerases (PPIases) such as FK506-binding protein 51 (FKBP51), FKBP52, and peptidylprolyl isomerase D (PPID, also known as Cyp40). These PPIase proteins play a crucial role in regulating the conformational cycle and chaperone activity of HSP90 39. In the HSP90α C-terminal inhibitor assay, HVH-2930 effectively inhibited the activity of C-terminal HSP90 by competitively interfering with the binding capability of the co-chaperone PPID, similar to the well-characterized C-terminal HSP90 inhibitor novobiocin (*p* < 0.0001, Figure 3F). Notably, unlike the N-terminal HSP90 inhibitors, neither HVH-2930 nor novobiocin exhibited any binding activity to the N-terminal region of HSP90α (NS, Figure 3G).

To explore the potential induction of the heat shock response (HSR) by HVH-2930, we conducted an immunocytochemical analysis for the subcellular localization of HSF1 and the expression levels of HSP70, HSP90 and HSP27. SKBR3 cells were exposed to HVH-2930, tanespimycin, and onalespib (300 nM, 24 h), followed by immunostaining for HSF1 and HSPs. Of particular note, we did not observe any elevation in HSF1 levels following the HVH-2930 challenge. In contrast, treatment with tanespimycin and onalespib led to a significant increase in nuclear accumulation of HSF1 (Figure 3H). Furthermore, HVH-2930 did not enhance the expression of HSP70, HSP90 and HSP27, whereas two N-terminal inhibitors markedly upregulated these protein levels (Figure 3I-J and Figure S7A-B). These findings were further confirmed by immunoblotting, which demonstrated that HVH-2930 did not impact the expression of HSPs in HER2-positive breast cancer cells (Figure S7C). Importantly, the expression and phosphorylation of HSF1 were also significantly downregulated by HVH-2930 treatment (*p* < 0.05, Figure 3K-L and Figure S8).

### HVH-2930 suppresses the HER2 signaling pathway by degrading HSP90 clients

**Figure 4:**
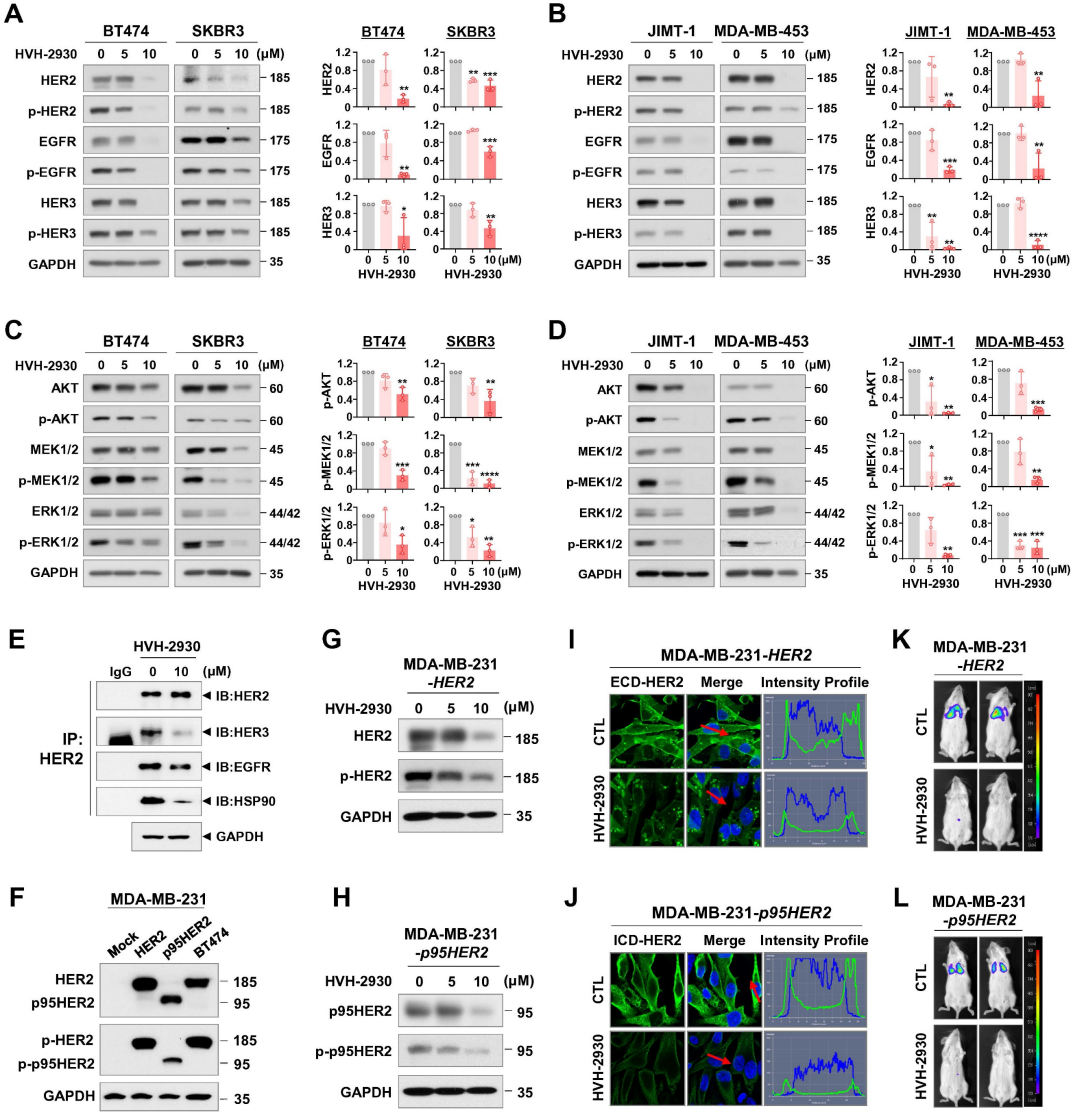
** HVH-2930 downregulates HSP90 client proteins in the HER2 signaling pathway.** (**A-B**) Immunoblot analyses of HER2, phospho-HER2 (Y1221/1222), EGFR, phospho-EGFR (Y1068), HER3 and phospho-HER3 (Y1289) protein expression in trastuzumab-sensitive (**A**) and -resistant cells (**B**) following exposure to HVH-2930 (5-10 μM, 72 h). Quantitative graphs represent the ratio of protein contents (*p < 0.05). (**C**-**D**) Effects of HVH-2930 on the expression of AKT, phospho-AKT (S473), MEK1/2, phospho-MEK1/2 (S217/221), ERK1/2 and phospho-ERK1/2 (T202/Y204) proteins in trastuzumab-sensitive (**C**, *p < 0.05) and -resistant cells (**D**, *p < 0.05). (**E**) Immunoblot analysis for HER2, HER3, EGFR, and HSP90 following immunoprecipitation with anti-HER2 antibody in JIMT-1 cells treated with HVH-2930 (10 μM, 24 h). IP, immunoprecipitation; IB, immunoblot; IgG, normal rabbit immunoglobulin G. (**F**) The expression levels of HER2, phospho-HER2, truncated p95HER and phospho-p95HER2 proteins by HER2- and p95HER2-overexpression in MDA-MB-231 cells. (**G-H**) Changes in the expression and phosphorylation of HER2 and p95HER2 in HER2- (**G**) and p95HER2-overexpressing cells (**H**) after treatment with HVH-2930 (5-10 μM, 72 h). (**I-J**) Immunocytochemical analysis for ECD- and ICD-HER2 in HER2- (**I**) and p95HER2-overexpressing cells (**J**) following exposure to HVH-2930 (10 μM, 24 h). (**K-L**) Influence of HVH-2930 on lung colonization using an experimental metastasis model *in vivo*. HER2- (**K**) and p95HER2-overexpressing MDA-MB-231 cells (**L**) were treated with HVH-2930 (10 μM) or control vehicle for 24 h, and then were injected into the tail veins of female NOD/SCID mice. Lung colonization from control or HVH-2930-treated mice was observed using an *in vivo* bioluminescence imaging (BLI) system.

As indicated by the public dataset analysis of gene expression described above, the HSP90 chaperone complex is more abundantly expressed in HER2-positive breast cancer than in non-malignant cells. It is also associated with an unfavorable prognosis, rendering it a promising target for pharmacological modulation. We next evaluated the potential inhibitory effects of HVH-2930 on HSP90 client proteins and downstream signaling pathways. Exposure to HVH-2930 (5-10 μM, 72 h) significantly reduced the total and phosphorylated levels of HER2 (Y1221/1222), EGFR (Y1068), and HER3 (Y1289) in both trastuzumab-sensitive and -resistant cells (*p* < 0.05, Figure 4A-B and Figure S9A). Notably, an immunoprecipitation assay with anti-HER2 antibodies revealed that HVH-2930 (10 µM, 24 h) reduced heterodimerization of HER2-HER3 and HER2-EGFR, as well as the interaction between HER2 and HSP90 in JIMT-1 cells (Figure 4E). Furthermore, the expression and phosphorylation of downstream signaling factors, including AKT (S473), MEK1/2 (S217/221), ERK1/2 (T202/Y204) and mTOR (S2448) were concomitantly downregulated (*p* < 0.05, Figure 4C-D, Figure S9B and S10A). Trastuzumab (10-100 μg/ml) did not affect the HER2 signaling pathway, as evidenced by the expression and phosphorylation of trastuzumab resistance-related molecules such as p95HER2, HER3, and AKT in JIMT-1 and MDA-MB-453 cells (Figure S11).

Accumulating studies have shown an inverse correlation between AKT activation and p27 levels, a cyclin-dependent kinase inhibitor. AKT phosphorylates p27 directly, causing its cytoplasmic sequestration. In contrast, inhibition of AKT facilitates the nuclear import of p27, which in turn regulates cell cycle progression and apoptosis by inhibiting the CDK2/cyclin E complex 40-42. HVH-2930 treatment led to an increase in p27 levels and its accumulation in the nucleus in trastuzumab-resistant JIMT-1 cells (Figure S10).

The presence of p95HER2 in HER2-positive breast cancer significantly impacts both prognosis and treatment response 43, 44. Approximately 30% of patients exhibit p95HER2 carboxy-terminal fragments, which are linked to poor clinical outcomes, heightened metastatic potential, and resistance to the standard HER2-targeted therapy, trastuzumab 45, 46. Targeting oncogenic p95HER2 has emerged as a promising strategy to improve treatment effectiveness and outcomes for HER2-positive breast cancer patients with this molecular profile. We confirmed that HVH-2930 treatment (5-10 μM, 72 h) effectively reduced the levels of full-length HER2 and truncated p95HER2, as well as their phosphorylated forms in HER2- and p95HER2-overexpressing MDA-MB-231 cells (Figure 4F-H). Immunofluorescence analysis showed dramatic reductions in the expression of extracellular domain (ECD)- and intracellular domain (ICD)-HER2 in the plasma membrane of HER2- and p95HER2-overexpressing cells following treatment with HVH-2930 (10 μM, 24 h), respectively (Figure 4I-J). We conducted further investigations to assess the influence of HVH-2930 on the propagation and lung colonization of HER2- and p95HER2-overexpressing cells in an *in vivo* experimental metastasis model. The MDA-MB-231-HER2 cells and MDA-MB-231-p95HER2 cells were treated with HVH-2930 (10 μM) or control vehicle for 24 h (viable cells > 97%, Figure S12). After normalizing the number of viable cells, the control or HVH-2930-treated cells (1×10^6^) suspended in 100 µl of culture medium were mixed and injected into the tail vein of 6-week-old female BALB/c nude mice. After 45 days, *in vivo* bioluminescence imaging (BLI) analysis revealed a striking reduction in the luminescence signal intensity, indicating an impediment in lung colonization caused by HVH-2930 challenge (Figure 4K-L). In addition, the kinetic analysis showed that exposure to HVH-2930 (1-20 μM, 72 h) reduced the migratory ability of JIMT-1 cells in a dose-dependent manner (Figure S13).

### HVH-2930 inhibits tumor growth of trastuzumab-resistant JIMT-1 xenografts

To confirm the physiological relevance of our *in vitro* findings, we assessed the effect of HVH-2930 on tumor growth and angiogenesis in a trastuzumab-resistant xenograft model by orthotopically injecting JIMT-1 cells (3×10^6^) into the fourth mammary fat pads of female BALB/c nude mice. Initially, to verify trastuzumab resistance, mice received trastuzumab starting with a loading dose of 4 mg/kg, followed by a weekly maintenance dose of 2 mg/kg, compared to a control solvent. There was no statistically significant difference in growth rates (NS, not significant, Figure S14A) or tumor mass between trastuzumab-treated tumors and their counterparts (NS, Figure S14B-C).

**Figure 5:**
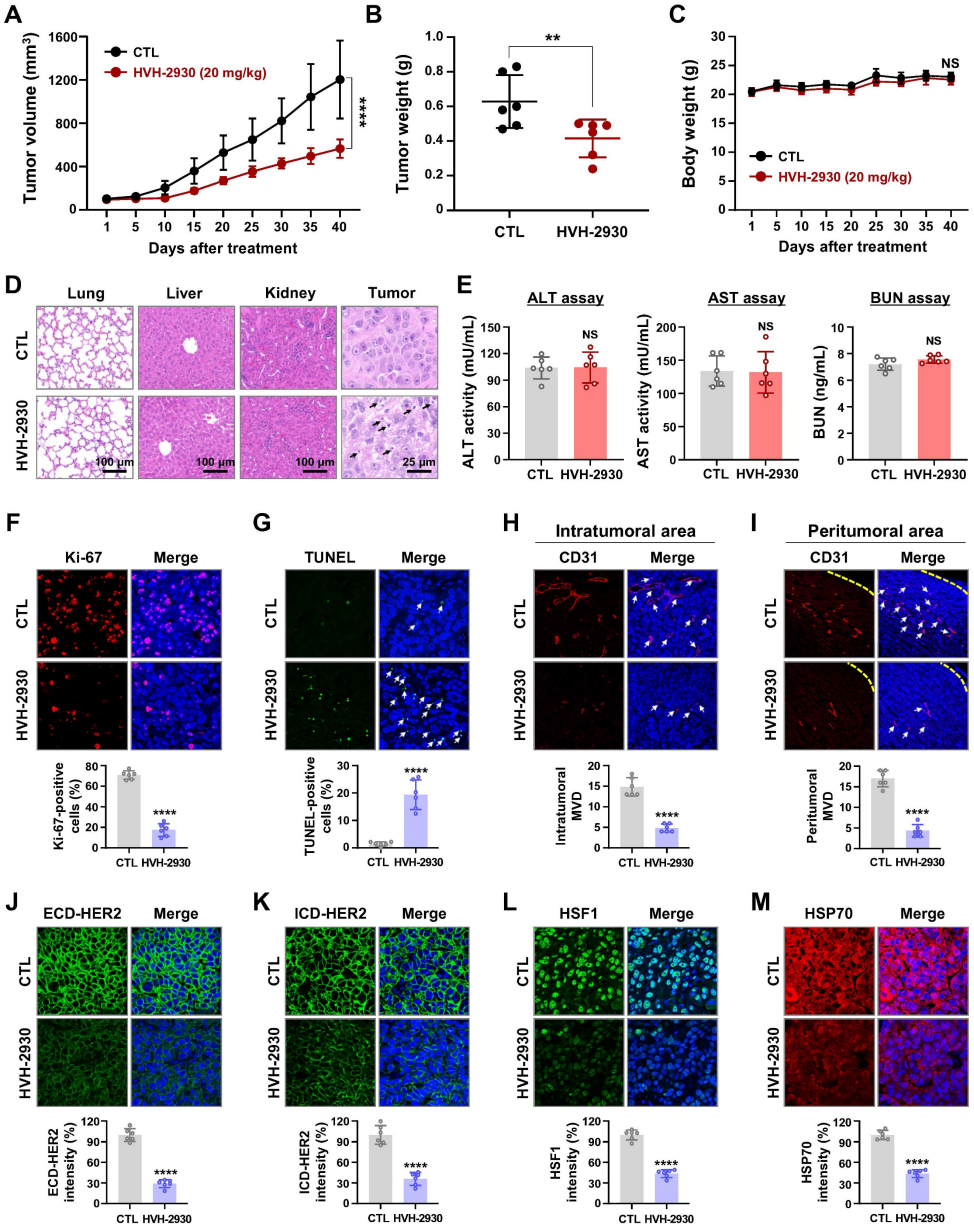
** HVH-2930 inhibits tumor growth in trastuzumab-resistant JIMT-1 xenografts.** (**A**-**C**) Effect of HVH-2930 on tumor growth *in vivo*. JIMT-1 cells (3×10^6^) were injected into the mammary fat pads of BALB/c nude mice (n = 6/each group). Following exposure to HVH-2930 (20 mg/kg, every other day) for 40 days, tumor growth (**A**, ****p < 0.0001), tumor weight (**B**, **p < 0.01) and body weight (**C**, NS, not significant) were evaluated. (**D**) Representative histological analysis of lung, liver, kidney, and tumor sections using hematoxylin and eosin (H&E) staining. (**E**) Effects of HVH-2930 on serum biochemical parameters of liver and kidney function. (**F-G**) Influence of HVH-2930 on Ki-67 expression and apoptosis *in vivo*. (**F**) Tumor tissue sections were immunostained for Ki-67 (red) and DAPI (blue). Percentage of Ki-67-positive cells (****p < 0.0001). (**G**) HVH-2930-induced apoptosis was determined by TUNEL assay (****p < 0.0001). (**H-I**) Effect of HVH-2930 on tumor angiogenesis, as determined by a microvessel density (MVD) assay. Tumor tissues were immunostained with a specific endothelial marker CD31 (red) and DAPI (blue). The number of CD31-positive microvessels in the intratumoral (**H**) and peritumoral areas (**I**) was quantified (****p < 0.0001). (**J-K**) Immunohistochemical analysis for ECD- (green,** J**, ****p < 0.0001) and ICD-HER2 (green, **K**, ****p < 0.0001) *in vivo*. (**L-M**) HVH-2930 administration resulted in significant downregulation of HSF1 (green, **L**, ****p < 0.0001) and HSP70 (red, **M**, ****p < 0.0001) *in vivo*.

To evaluate the anti-tumor efficacy of HVH-2930 in the trastuzumab-resistant xenograft model, the mice were subjected to either HVH-2930 treatment (20 mg/kg, every other day) or received a control vehicle (1:9, DMSO:saline). HVH-2930 administration significantly retarded tumor growth (*p* < 0.0001, Figure 5A) and reduced tumor weight (*p* < 0.01, Figure 5B) without detrimental effects on body weight (NS, Figure 5C). Histopathological analyses revealed increased cell shrinkage and nuclear condensation in HVH-2930-treated tumor tissues compared to controls, whereas no histological alterations were observed in the kidney, liver, and lung tissues (Figure 5D). We further evaluated hepatorenal toxicity by measuring the levels of aspartate aminotransferase (AST), alanine aminotransferase (ALT) and blood urea nitrogen (BUN) on serum samples from the animals. The results showed no significant changes between the control and treatment groups, indicating that HVH-2930 does not adversely affect liver or kidney function (NS, Figure 5E).

The antitumor effect of HVH-2930 was accompanied by a marked reduction in Ki-67-positive cells (*p* < 0.0001, Figure 5F) and a significant increase in apoptosis (*p* < 0.0001, Figure 5G). To further assess the impact of HVH-2930 on tumor angiogenesis, a microvessel density (MVD) assay was conducted using the endothelial-specific marker CD31 47. HVH-2930 administration significantly suppressed the number of CD31-positive vessels in both intratumoral (*p* < 0.0001, Figure 5H) and peritumoral areas (*p* < 0.0001, Figure 5I). Consistent with *in vitro* observations, HVH-2930 significantly downregulated the expression of both full-length HER2 (*p* < 0.0001, Figure 5J) and ICD-HER2 (*p* < 0.0001, Figure 5K). Furthermore, HVH-2930 administration effectively inhibited HSF1 activity, as evidenced by decreased nuclear HSF1 signal intensity (*p* < 0.0001, Figure 5L) and downregulation of its downstream mediator HSP70 (*p* < 0.0001, Figure 5M).

### HVH-2930 attenuates CSC-like properties in HER2-positive breast cancer cells

**Figure 6:**
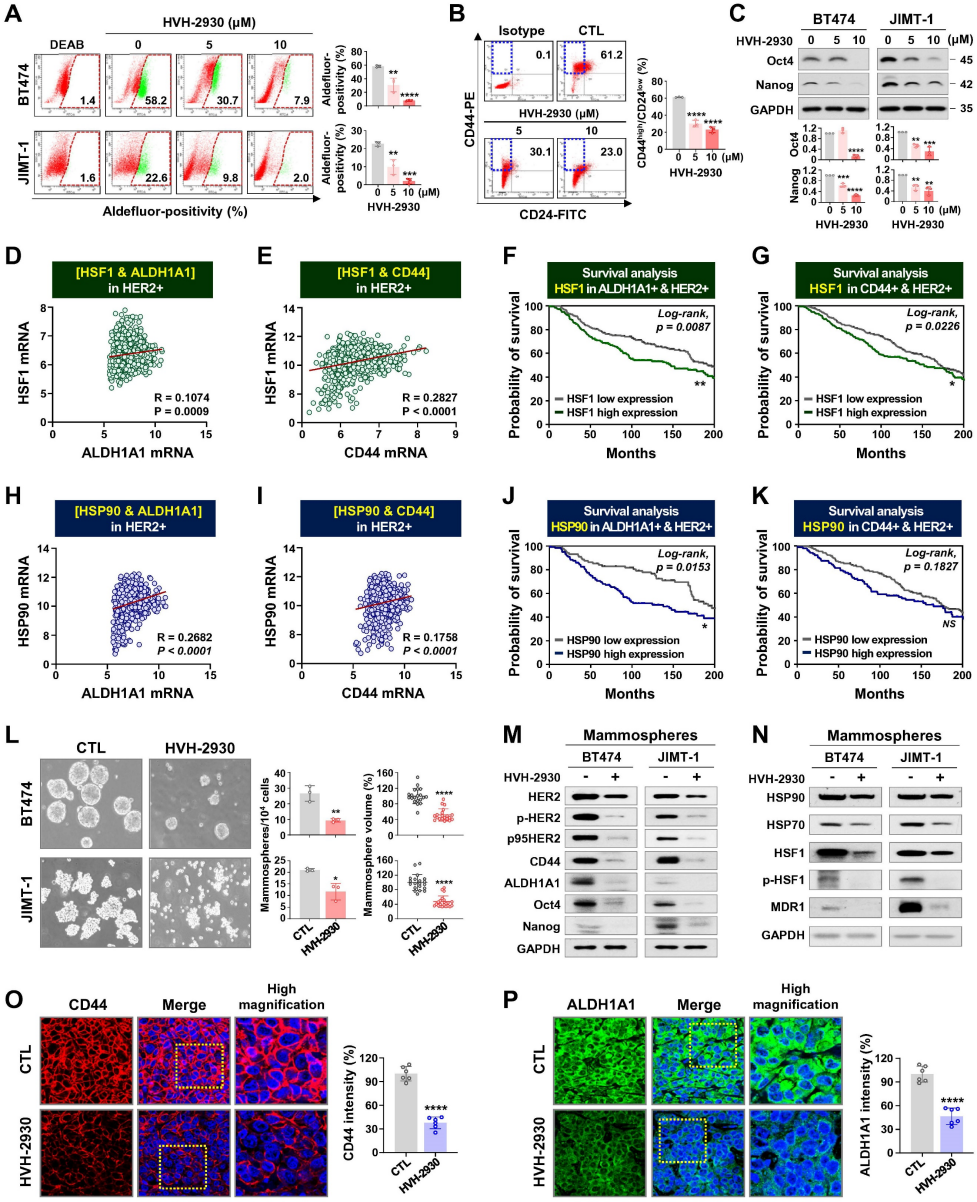
** HVH-2930 impairs CSC-like properties by disrupting the HSP90-HSF1-HER2 axis.** (**A-B**) Effect of HVH-2930 on CSC-like characteristics. Cells were treated with HVH-2930 (5-10 μM, 72 h). Aldefluor-positivity in BT474 and JIMT-1 cells (**A**, **p < 0.01) and CD44^high^/CD24^low^ populations in JIMT-1 cells (**B**, ****p < 0.0001) determined by flow cytometry. (**C**) Changes in the expression of Nanog and Oct4 protein in BT474 and JIMT-1 cells following exposure to HVH-2930 (5-10 μM, 72 h). Quantitative graphs represent the ratio of Nanog/GAPDH and Oct4/GAPDH (**p < 0.01). (**D-E**) Correlation of mRNA levels between HSF1 and either ALDH1A1 (**D**, ***p = 0.0009) or CD44 (**E**, ****p < 0.0001) in breast cancer patients with high HER2 expression from the METABRIC dataset. (**F-G**) Kaplan-Meier survival curves depict the overall survival of breast cancer patients with low or high HSF1 mRNA expression correlated to either ALDH1A1-high/HER2-high (**F**, **p = 0.0087) or CD44-high/HER2-high expression (**G**, *p = 0.0226). (**H-I**) Correlation of mRNA expression between HSP90 and both ALDH1A1 (**H**, ****p < 0.0001) and CD44 (**I**, ****p < 0.0001**)**. (**J-K**) Overall survival according to low or high mRNA expression between HSP90 and ALDH1A1-high/HER2-high (**J**, *p = 0.0153) and between HSP90 and CD44-high/HER2-high (**K**, *p* = 0.1827). (**L**) Effect of HVH-2930 on mammosphere formation. BT474 (3×10^5^) and JIMT-1 (1.5×10^5^) cells were cultured in serum-free suspension conditions in the presence or absence of HVH-2930 (10 μM) for 4 and 7 days, respectively. Number and volume of mammospheres quantified by optical microscopy (*p < 0.05). (**M-N**) Changes in expression of HER2, p95HER2, phospho-HER2, CD44, ALDH1A1, Oct4, Nanog, HSP90, HSP70, HSF1, phospho-HSF1 (S326) and MDR1 proteins in BT474- and JIMT-1-mammospheres after HVH-2930 treatment. (**O-P**) Immunohistochemical analyses for CD44 (red, **O**, ****p < 0.0001) and ALDH1A1 (green, **P**, ****p < 0.0001) with DAPI (blue) in JIMT-1 xenograft tumors following exposure to HVH-2930 (20 mg/kg, 40 days).

CSCs are a major contributor to trastuzumab resistance in HER2-positive breast cancer, fostering recurrence and metastatic spread 18, 21. HSP90 plays a crucial role in maintaining the CSC phenotype and stability of pluripotent transcription factors such as Nanog and Oct4, referred to as client proteins 24, 48. This indicates that targeting HSP90 could effectively impair the stemness and pluripotency of cancer cells. In our previous report, we observed simultaneous upregulation of the HSP90 chaperone complex and HER2 in CSC-enriched populations 24. Exposure to HVH-2930 (5-10 μM, 72 h) not only induced a dose-dependent reduction in ALDH1 activity in both BT474 and JIMT-1 cells (*p* < 0.01, Figure 6A); it also led to a significant decrease in JIMT-1 CD44^high^/CD24^low^ subpopulations (*p* < 0.0001, Figure 6B). HVH-2930 (5-10 μM, 72 h) resulted in a marked downregulation in the expression of Oct4, Nanog, CD44 and ALDH1A1 (*p* < 0.01, Figure 6C and Figure S15B). In contrast, N-terminal HSP90 inhibitors, tanespimycin (100 nM) or onalespib (500 nM, 72 h) in JIMT-1 cells led to a modest downregulation of Oct4 and ALDH1A1 expression (Figure S15). These findings suggest the superior efficacy of HVH-2930 in targeting CSC traits compared to N-terminal HSP90 inhibitors.

Utilizing the METABRIC data cohort, we conducted an analysis of the correlation between CSC markers and the HSP90 chaperone complex. A substantial positive correlation was observed between HSF1 and either ALDH1A1 (*p* = 0.0009, Figure 6D) or CD44 (*p* < 0.0001, Figure 6E) in breast cancer patients with high HER2 expression. Notably, elevated HSF1 mRNA levels in patients with high expression of both ALDH1A1 and HER2 or both CD44 and HER2 were associated with relatively worse overall survival (Figure 6F-G, respectively). These findings are consistent with a positive correlation between HSP90 and either ALDH1A1 (*p* < 0.0001, Figure 6H) or CD44 (*p* < 0.0001, Figure 6I), as well as poor overall survival in patients with concurrently high HSP90/ALDH1A1/HER2 or HSP90/CD44/HER2 expression (Figure 6J-K).

Under anchorage-independent serum-free culture conditions, BT474 and JIMT-1 cells displayed the ability to form mammospheres with highly enriched CSC-like populations. By days 4 and 7, respectively, control BT474- and JIMT-1-mammospheres formed regular three-dimensional spheres, while treatment with HVH-2930 (10 μM) significantly suppressed sphere-forming ability, as evidenced by reduced numbers and volumes of mammospheres (*p* < 0.05, Figure 6L). In HVH-2930-treated mammospheres, the expression of CD44, ALDH1A1, Oct4, and Nanog was dramatically diminished, accompanied by marked downregulation of HER2, phospho-HER2, and p95HER2 expression (Figure 6M). HVH-2930 also reduced the expression of HSP90, HSP70, HSF1 and phospho-HSF1, coinciding with the downregulation of MDR1 (also known as p-glycoprotein) levels, a transcriptional target of HSF1 (Figure 6N). To further confirm the effect of HVH-2930 on CSC-like characteristics, we performed immunohistochemical analysis for CD44 and ALDH1A1 in JIMT-1 xenograft tumors. Consistent with the *in vitro* data, the levels of two CSC markers were significantly suppressed by HVH-2930 administration (*p* < 0.0001, Figure 6O-P, respectively). This finding suggests that HVH-2930 could be more effective against trastuzumab resistance in HER2-positive breast cancer by targeting CSC-like traits.

### Effect of HVH-2930 and paclitaxel combination treatment on JIMT-1 xenograft tumors

**Figure 7:**
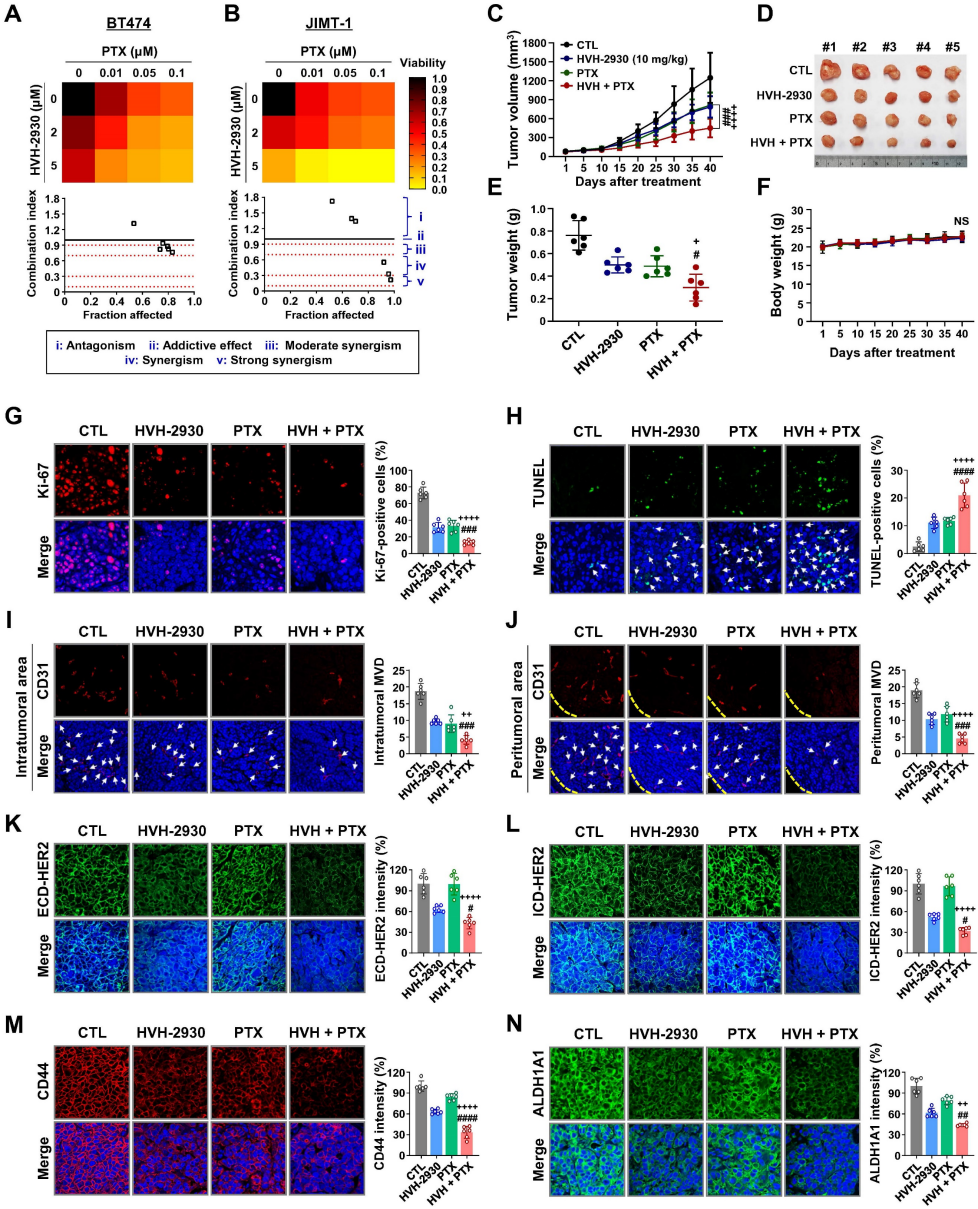
** Effect of HVH-2930 and paclitaxel (PTX) combination treatment on trastuzumab-resistant JIMT-1 xenografts.** (**A**-**B**) The co-treatment effect of HVH-2930 and PTX on cell viability in BT474 (**A**) and JIMT-1 cells (**B**). Cells were treated at the indicated concentrations of HVH-2930 (2-5 μM) and PTX (0.01-0.1 μM) for 72 h and cell viability was assessed by MTS assay. Color intensity represents relative cell viability compared with DMSO control. The bottom panel shows combination indices for HVH-2930 and PTX in each cell line. The combination index (CI) was used to quantify synergism or antagonism, where CI < 1, = 1, and > 1 indicate synergistic, additive or antagonistic effects, respectively. (**C-F**) Effect of HVH-2930 and PTX combination treatment on tumor growth *in vivo*. JIMT-1 xenografted mice received intraperitoneal administrations of vehicle, HVH-2930 (10 mg/kg·BW, every other day), PTX (4 mg/kg·BW, once a week), or a combination of the two compounds (n = 5/each group). After 40 days of administration, tumor growth (**C**, ###*p* < 0.001, HVH-2930 only vs combination; ++++*p* < 0.0001, PTX only vs combination), tumor burden (**D**), tumor weight (**E**, +*p* < 0.05, #*p* < 0.05) and body weight (**F**, NS, not significant) were evaluated. (**G-H**) Influence of HVH-2930 and PTX combination treatment on Ki-67 expression and apoptosis *in vivo*. (**G**) Tissue sections were immunostained for Ki-67 (red) with DAPI (blue), and Ki-67-positive cells were counted (###*p* < 0.001, ++++*p* < 0.0001). (**H**) Apoptosis induction was measured by TUNEL assay (green) and nuclei were counterstained with DAPI (blue). The graph shows the percentage of TUNEL-positive cells (####*p* < 0.0001, ++++*p* < 0.0001). (**I-J**) Tumor tissues were immunostained with CD31 (red) and DAPI (blue) and CD-31-positive microvessels in the intratumoral (**I**, ###*p* < 0.001, ++*p* < 0.01) and peritumoral areas (**J**, ###*p* < 0.001, ++++*p* < 0.0001) were quantified. (**K-L**) Immunohistochemical analysis for the ECD-HER2 (green, **K,** #*p* < 0.05, ++++*p* < 0.0001) and ICD-HER2 (green, **L,** #*p* < 0.05, ++++*p* < 0.0001) *in vivo*. (**M-N**) Effect of combination of HVH-2930 and PTX on the expression of CSC markers CD44 and ALDH1A1 *in vivo*. Fluorescence intensities of CD44 (red, **M**, ####*p* < 0.0001, ++++*p* < 0.0001) and ALDH1A1 (green, **N**, ##*p* < 0.01, ++*p* < 0.01) were quantified.

Treatment with HER2-targeted therapeutics plus taxane compounds has been shown to be highly effective for HER2-positive breast cancer 49, 50. Paclitaxel (PTX) is one of the most widely used chemotherapeutic agents in various combinations due to its effectiveness and broad spectrum of antitumor activity 51. BT474 and JIMT-1 cells were treated with HVH-2930 (0-5 μM) and/or PTX (0-0.1 μM) for 72 h. HVH-2930 at 5 μM in combination with PTX (0.01-0.1 μM) exhibited significant synergistic antiproliferative activity compared to either agent alone in JIMT-1 cells, but only moderate synergism was observed in BT474 cells (Figure 7A-B and Figure S16). On the other hand, the combination of HVH-2930 (0-5 μM, 72 h) and trastuzumab (0-100 μg/ml, 72 h) failed to induce a synergistic antiproliferative effect in both trastuzumab-sensitive and -resistant cells, compared to the individual treatments with HVH-2930 or trastuzumab (Figure S17).

We further investigated the *in vivo* antitumor effects of HVH-2930 and PTX using a xenograft model bearing JIMT-1 tumors. Mice were intraperitoneally administrated with either a vehicle, HVH-2930 (10 mg/kg, every other day), PTX (4 mg/kg, once a week), or a combination regimen for 40 days. When administrated alone and in combination, both regimens demonstrated significant inhibitory effects on tumor growth compared to the control group (Figure 7C-E). Treatment with HVH-2930 alone resulted in a tumor growth inhibition rate of 36.93% (*p* < 0.001, vs control) and PTX alone yielded 34.77% inhibition (*p* < 0.0001, vs control). In contrast, the combination of HVH-2930 and PTX led to significantly greater inhibition of tumor growth than either agent alone, with an inhibition rate of 63.75% (*p* < 0.0001, vs HVH-2930-treated group; *p* < 0.001, vs PTX-treated group). Notably, no instances of body weight loss or organ toxicity were observed during the treatment period in any of the groups (Figure 7F, Figure S18 and S19, respectively).

A significant reduction in proliferative Ki-67-positive cells was observed following the administration of HVH-2930 in combination with PTX (*p* < 0.001, Figure 7G), accompanied by a marked increase in apoptotic cell death in the combination group (*p* < 0.0001, Figure 7H). In addition, the number of CD31-positive blood vessels was substantially decreased in both peritumoral and intratumoral areas in the combination group, with a significant difference to the alone groups (*p* < 0.01, Figure 7I-J). In the combination group, a significant reduction in the expression of both ECD- and ICD-HER2 was observed when compared to the groups treated with either agent alone (*p* < 0.05, Figure 7K-L, respectively). Furthermore, the combination of HVH-2930 and PTX resulted in a marked decrease in the expression of CD44 (*p* < 0.0001, Figure 7M) and ALDH1A1 (*p* < 0.01, Figure 7N).

## Discussion

While the role of HSP90 in trastuzumab resistance has been previously reported, the clinical failure of N-terminal domain-targeted inhibitors has been attributed to poor bioavailability, off-target effects, and undesirable induction of the HSR, which promotes oncogenic signaling 6, 10, 52. In order to address these issues, we have rationally-designed HVH-2930 to target the C-terminal domain.

To elucidate the interaction between HVH-2930 and HSP90, we employed computational docking and SPR analysis. The results show that HVH-2930 fits into the predicted ATP-binding pocket and interface cavity of the hHSP90 homodimer in the C-terminal domain, stabilizing its open conformation and hindering ATP binding. Notably, SPR analysis confirmed the dose-dependent binding of HVH-2930 to HSP90α with higher affinity than novobiocin, a widely-studied C-terminal HSP90 inhibitor.

Inhibition of HSP90 by classical HSP90 inhibitors, such as alvespimycin (17-DMAG), tanespimycin (17-AAG) and onalespib (AT13387), increases the levels of HSP70, HSP90, and HSF1 53, 54. The HSR is a robust defense mechanism that impedes the pro-apoptotic activity of N-terminal HSP90 inhibitors, fostering drug resistance. HSP70 represents an obstacle to apoptotic signaling, impeding the translocation of Bax into the mitochondria and directly binding with Apaf-1. This hinders the formation of apoptosome complex involving caspase-9 and cytochrome c 55, 56. Therefore, higher doses are necessary for these agents to achieve anti-tumor effects, posing a potential risk of dose-limiting toxicities.

It is noteworthy that HVH-2930 exerts anti-tumor effects in both trastuzumab-sensitive and -resistant HER2-positive breast cancer cells without triggering the HSR. HVH-2930 induces mitochondria-dependent apoptosis by promoting oxidative stress and increased active-p18Bax, the release of cytochrome c and activation of caspases, and the depletion of ATP synthesis, indicating disruption of mitochondrial integrity. Importantly, HVH-2930 exhibits minimal cytotoxicity in non-malignant cells, with IC50 values ranging from 200 to 1000 times lower than those of the first-generation N-terminal HSP90 inhibitors tanespimycin and second-generation onalespib. In our prior investigation, blood analysis of alvespimycin, tanespimycin and onalespib revealed elevated ALT and AST levels, as evidenced by hepatotoxicity 27. Clinical and preclinical studies further indicated significant nephrotoxicity associated with these inhibitors 27, 57-59. Notably, HVH-2930 administration exhibited no adverse effects on markers of liver or kidney health *in vivo*.

HSF1 plays a multifaceted role in carcinogenesis, being vital for cancer adaptation and survival under challenging pathophysiological conditions like hypoxia, acidosis, ATP deprivation and nutritional changes 60-62. Analysis of HER2-positive breast cancer datasets reveals a positive correlation between HSF1 and components of the HSP chaperone complex, including HSP90 and HSP70, with simultaneous overexpression linked to poorer clinical outcomes. In the HER2 signaling pathway, HSF1 acts as a crucial determinant, conferring a comprehensive range of pro-survival effects and facilitating chemoresistance 63, 64. Mechanisms underlying trastuzumab resistance involve HER2/HER3 and HER2/EGFR interactions, coupled with hyperactivation of AKT signaling 65, 66. AKT directly interacts with HSF1, phosphorylating the Ser326 residue, which induces its trimerization and translocation to the nucleus 67, 68. Treatment with HVH-2930 was observed to alleviate HSF1 phosphorylation, thereby preventing nuclear accumulation and HSP70 upregulation in HER2-positive breast cancer cells, without inducing HSF1 transcriptional activity.

The METABRIC data cohort identified a significant correlation between overexpression of CD44/ALDH1A1 and HSP90 or HSF1 in patients with HER2-positive breast cancer, which was associated with relatively poorer overall survival. In particular, CD44 is a prominent cancer stem cell marker and trastuzumab resistance factor, playing a pivotal role in regulating neovascularization and metastatic spread via the secretion of proteolytically active MMP-9 69, 70. Additionally, CD44 interacts with a cytoskeletal protein complex comprising ankyrin, ERM proteins (ezrin, radixin, and moesin), and actin. This interaction orchestrates the dynamic regulation of the cancer cell cytoskeleton and specialized structures essential for migration 71, 72. Marked reductions in CD44 expression were observed in trastuzumab-resistant CSC subpopulations in the presence of HVH-2930. Furthermore, cell migration analysis revealed that HVH-2930 dose-dependently impairs the migratory capability of JIMT-1 cells *in vitro*. It is conceivable that downregulation of CD44 may contribute to the inhibition of migratory ability, thereby preventing metastasis.

MDR1 upregulation is a significant marker of aggressive and refractory tumors, showing a substantial correlation with shorter overall survival and high heterogeneity 73. MDR1 facilitates the efflux of chemotherapy agents like doxorubicin and paclitaxel, contributing to the dissemination of tumor cells to distant organs and triggering recurrence post-treatment 74-76. Our previous study revealed a notable upregulation of MDR1 in mammospheres and ALDH1-positive cells, representing a stem-like subpopulation 73. Numerous phase I/II clinical studies of 17-AAG have demonstrated that its use as a single agent does not yield significant clinical benefit 77. Evidence suggests that exposure to ansamycin benzoquinones, including geldanamycin and 17-AAG, heightens MDR1 expression, leading to resistance against HSP90 inhibitors 78. This phenomenon may at least in part explain the failure of N-terminal HSP90 inhibitors in clinical trials. The MDR1 gene, harboring a heat shock element (HSE) in its promoter region, activates MDR1 expression upon HSF1 activation during the heat shock response 79. The C-terminal HSP90 inhibitor HVH-2930 effectively suppressed MDR1 expression in CSC-enriched mammospheres by inhibiting HSF1 activity, thus contributing to the elimination of the CSC population in trastuzumab resistant settings.

## Conclusion

**Figure 8:**
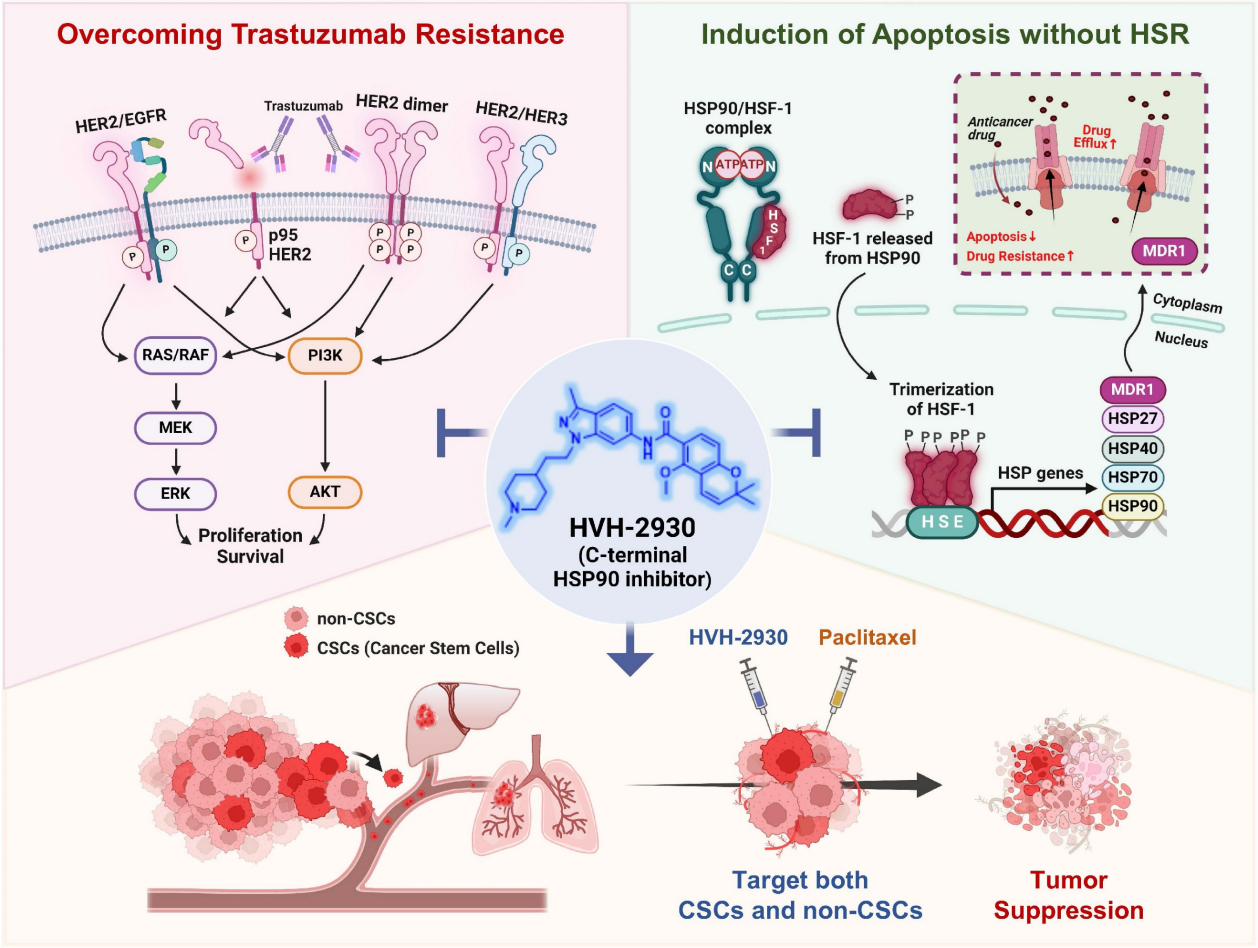
** Hypothetical model illustrating the multiple actions of HVH-2930 on the HER2 signaling pathway and the HSP90-HSF1 axis in trastuzumab-resistant HER2-positive breast cancer.** HVH-2930 exerts potent antitumor activity by addressing trastuzumab resistance in HER2-positive breast cancer without triggering the HSR. As a novel C-terminal HSP90 inhibitor, HVH-2930 downregulates full-length HER2, p95HER2, and other HER family members. It also attenuates heterodimerization of HER2/HER3 or HER2/EGFR, leading to the disruption of multiple survival pathways, including PI3K/AKT and MEK/ERK signaling. These phenomena are associated with the direct modulation of the HSP90 chaperone complex without HSF1 activation. Furthermore, the impairment of the HSP90-HSF1 axis by HVH-2930 leads to the suppression of CSC-like properties and the downregulation of MDR1, a multidrug efflux pump. In trastuzumab-resistant xenograft models, HVH-2930 retards tumor growth and angiogenesis, and impedes metastatic ability. Notably, the combination of HVH-2930 and paclitaxel eliminates both rapidly proliferating cancer cells and CSCs, resulting in a synergistic increase in antitumor efficacy.

HVH-2930, a novel C-terminal HSP90 inhibitor, exhibits significant anticancer activity in trastuzumab-resistant HER2-positive breast cancer without inducing the heat shock response. It instead induces mitochondria-dependent apoptosis, attenuates CSC-like properties, and targets the HER2 signaling pathway. This is accompanied by the downregulation of HER2 and p95HER2, critically disrupting heterodimerization of HER2 family members. Additionally, key signaling molecules including AKT, MEK1/2, and ERK1/2 are downregulated, leading to the simultaneous inhibition of multiple survival signals. We hypothesize that these cumulative effects elicit the potent suppression of angiogenesis and tumor growth in trastuzumab-resistant xenograft mice. The observed synergy in combination with paclitaxel highlights a multifaceted approach for improving treatment outcomes in HER2-positive breast cancer (Figure 8, hypothetical model). Further investigation is warranted to fully establish the therapeutic potential of HVH-2930.

## Supplementary Material

Supplementary methods and figures.

## Abbreviations

ALDH1: aldehyde dehydrogenase 1
AKT: protein kinase B
ATP: adenosine triphosphate
AST: aspartate aminotransferase
ALT: alanine aminotransferase
BLI: bioluminescent imaging
BUN: blood urea nitrogen
CSC: cancer stem cell
CD31: cluster of differentiation 31
CD44: cluster of differentiation 44
DEAB: diethylamino-benzaldehyde
ECD: extracellular domain
EGFR: epidermal growth factor receptor
ERK: extracellular signal-regulated kinase
GAPDH: glyceraldehyde-3-phosphate dehydrogenase
HER2: human epidermal growth factor receptor 2
HER3: human epidermal growth factor receptor 3
HSR: heat shock response
HSF1: heat shock factor 1
HSP70: heat shock protein 70
HSP90: heat shock protein 90
H&E: hematoxylin and eosin
IP: Immunoprecipitation
ICD: intracellular domain
MEK: mitogen-activated protein kinase
METABRIC: The Molecular Taxonomy of Breast Cancer International Consortium
NANOG: nanog homeobox
OCT4: octamer-binding transcription factor 4
PARP: poly (ADP-ribose) polymerase
PI3K: phosphatidylinositol-4,5-bisphosphate 3-kinase
PTX: paclitaxel
ROS: reactive oxygen species
SPR: surface plasmon resonance
TCGA: the cancer genome atlas
