# Supplementary material for: The HSP90 inhibitor HVH-2930 exhibits potent efficacy against trastuzumab-resistant HER2-positive breast cancer
Source: Theranostics. 2024 Mar 31;14(6):2442–63. doi: 10.7150/thno.93236 (PMC11024854; doi:10.7150/thno.93236)
Supplement: Supplementary file 1 — Supplementary methods and figures. [file thnov14p2442s1.pdf]

Supplementary information 1

The HSP90 inhibitor HVH-2930 exhibits potent efficacy against trastuzumab-resistant HER2-positive breast cancer

Minsu Park[1,2,*], Eunsun Jung[1,3,*], Jung Min Park[1,2], Soeun Park[1,2], Dongmi Ko[1,2], Juyeon Seo[1,2], Seongjae Kim[1,2], Kee Dal Nam[1,3], Yong Koo Kang[1,3], Lee Farrand[4], Van-Hai Hoang[5], Cong-Truong Nguyen[6], Minh Thanh La[7], Gibeom Nam[8], Hyun-Ju Park[8], Jihyae Ann[7,†], Jeewoo Lee[7,†], Yoon-Jae Kim[1,2,3,†], Ji Young Kim[1,3,†] and Jae Hong Seo[1,2,3,†]

Design of HVH-2930 as HSP90 Inhibitors by Bioisosteric approach

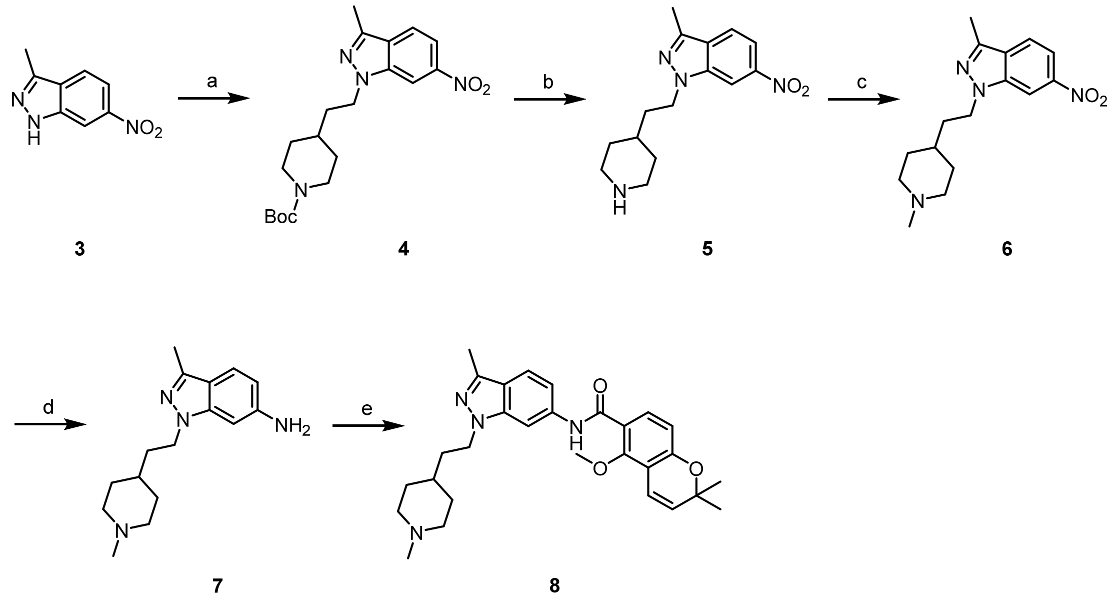

Scheme 1. Synthesis of HVH-2930.

Conditions and reagents: (a) *tert*-butyl 4-(2-bromoethyl)piperidine-1-carboxylate, Cs_2CO_3, DMF, r.t., 18 h; (b) TFA, DCM, r.t., 3 h; (c) $(HCHO)_n$, $NaBH_4$, CF_3CH_2OH, r.t., 2 h; (d) 10% Pd/C, H_2 (g), MeOH, r.t. 3 h; (e) 5-methoxy-2,2-dimethyl-2H-chromene-6-carboxylic acid, EDC·HCl, HOBt, TEA, DCM, r.t, 15 h.

Experimental section

General. All chemical reagents were commercially available. Silica gel column chromatography was performed on silica gel 60, 230–400 mesh, Merck. Reaction progress was monitored by thin layer chromatography silica gel 60 F_{254}, 0.5 mm, Merck. ^1H spectra were

recorded on a Jeol Resonance ECZ 400S at 400 MHz. Chemical shifts are reported in ppm units with Me_4Si as a reference standard. The purity of representative compound was measured by using high-performance liquid chromatography (HPLC) on Agilent 1120 Compact LC (G4288A) instrument using Agilent TC-C18 column (4.6 mm × 250 mm, 5 μm). Detection was performed at 290 nm as following condition; temperature = 25 °C, flow rate = 0.7 mL/min. The eluent system consisted of 70% eluent A (MeOH with 0.1% formic acid, HPLC grade) and 30% eluent B (H_2O with 0.1% formic acid, HPLC grade).

***tert*-Butyl 4-(2-(3-methyl-6-nitro-1H-indazol-1-yl)ethyl)piperidine-1-carboxylate (4).**

To a solution of **3** (7.18 g, 40.75 mmol) in DMF (50 mL), *tert*-butyl 4-(2-bromoethyl)piperidine-1-carboxylate (13.10 g, 44.83 mmol) and cesium carbonate (19.91 g, 61.13 mmol) was added and stirred for 15 h at room temperature. After completion, the reaction mixture was quenched with H_2O and extracted with EtOAc. The combined organic layer was washed with brine, dried over $MgSO_4$, filtered and concentrated *in vacuo*. The crude mixture was purified by column chromatography (hexane/ethyl acetate = 1:4) to give **4** as a yellow solid (10.1 g, 64%). ^{1}H NMR (400 MHz, DMSO-d_6) δ 8.65 (dd, J = 1.9, 0.7 Hz, 1H), 7.92 (dd, J = 8.9, 0.7 Hz, 1H), 7.86 (dd, J = 8.8, 1.9 Hz, 1H), 4.47 (t, J = 7.2 Hz, 2H), 3.92-3.81 (m, 2H), 2.67-2.53 (m, 2H), 2.51 (s, 3H), 1.73 (q, J = 7.1 Hz, 2H), 1.68-1.62 (m, 2H), 1.44-1.36 (m, 1H), 1.34 (s, 9H), 1.10-0.90 (m, 2H).

3-Methyl-6-nitro-1-(2-(piperidin-4-yl)ethyl)-1H-indazole (5).

To the solution of **4** (10.1 g, 26.00 mmol) in DCM (20 mL), TFA (5 mL) was added and stirred at room temperature for 3 h. After completion, solvent was evaporated and the residue was basified by 10% aqueous NaOH, and then extracted with EtOAc. The combined organic layer was washed with water, dried over Na_2SO_4 and concentrated *in vacuo* to yield **5** as brown solid (7.49 g), which was used for the next step without further purification. ^{1}H NMR (400 MHz, $CDCl_3$) δ 8.27 (d, J = 1.8 Hz, 1H), 7.96 (dd, J = 8.8, 1.8 Hz, 1H), 7.73 (d, J = 8.8 Hz, 1H), 4.41 (t, J = 7.4 Hz, 2H), 3.17-3.12 (m, 2H), 2.65 – 2.57 (m, 2H), 2.60 (s, 3H), 1.89-1.86 (m, 2H), 1.82-1.75 (m, 2H), 1.50-1.41 (m, 1H), 1.39-1.26 (m, 2H).

3-Methyl-1-(2-(1-methylpiperidin-4-yl)ethyl)-6-nitro-1H-indazole (6).

To a solution of **5** (7.49 g, 25.98 mmol) in trifluoroethanol (50 mL), paraformaldehyde (2.34 g, 77.94 mmol) and sodium borohydride (2.95 g, 77.94 mmol) were added and stirred at room

temperature for 2 h. After completion, solvent was removed *in vacuo*, and the crude mixture was diluted with EtOAc. The combined mixture was washed with water, dried over Na_2SO_4 and concentrated *in vacuo*. The mixture was purification by column chromatography on silica gel with MeOH :CH_2Cl_2 as eluent to yield **6** as a yellow solid (7.30 g, 93%). ^{1}H NMR (400 MHz, $CDCl_3$) δ 8.27 (d, *J* = 1.5 Hz, 1H), 7.96 (dd, *J* = 8.9, 1.9 Hz, 1H), 7.73 (d, *J* = 8.8 Hz, 1H), 4.41 (t, *J* = 7.2 Hz, 2H), 3.13-3.02 (m, 2H), 2.59 (s, 3H), 2.42 (s, 3H), 2.19-2.13 (m, 2H), 1.95-1.89 (m, 2H), 1.86-1.80 (m, 2H), 1.69-1.56 (m, 2H), 1.39-1.27 (m, 1H).

3-Methyl-1-(2-(1-methylpiperidin-4-yl)ethyl)-1H-indazol-6-amine (7)

To a solution of **6** in methanol, 10% Pd/C (730 mg) was slowly added and stirred under H_2 (g) at room temperature for 3 h. After completion, the mixture was filtered through celite® and washed with methanol. The filtrate was concentrated *in vacuo* to yield **7** as light red solid (6.25 g, 95%), which was used for the next step without further purification. ^{1}H NMR (400 MHz, CD_3OD) *δ* 7.35 (d, *J* = 8.6 Hz, 1H), 6.57 (dd, *J* = 8.5, 1.8 Hz, 1H), 6.55 (d, *J* = 1.8 Hz, 1H), 4.17 (t, *J* = 7.1 Hz, 2H), 2.84-2.77 (m, 2H), 2.40 (s, 3H), 2.21 (s, 3H), 1.96-1.89 (m, 2H), 1.74-1.68 (m, 4H), 1.28-1.22 (m, 3H).

5-Methoxy-2,2-dimethyl-N-(3-methyl-1-(2-(1-methylpiperidin-4-yl)ethyl)-1H-indazol-6-yl)-2H-chromene-6-carboxamide (8).

To a solution of **7** (6.25 g, 22.94 mmol) in CH_2Cl_2 (50 mL), 5-methoxy-2,2-dimethyl-2*H*-chromene-6-carboxylic acid (5.91 g, 25.24 mmol), EDC·HCl (6.60 g, 34.42 mmol), HOBt (4.65 g, 34.42 mmol) and triethylamine (6.40 mL, 45.89 mmol) were added and stirred at room temperature for 15 h. After completion, the mixture was extracted with EtOAc and the combined organic layer was washed with water, dried over Na_2SO_4 and concentrated *in vacuo*. The residue was purification by silica gel column chromatography dichloromethane with 8% methanol to afford **8** as a white solid (6.60 g, 59%). ^{1}H NMR (400 MHz, $CDCl_3$) *δ* 9.92 (s, 1H), 8.32 (d, *J* = 1.6 Hz, 1H), 7.98 (d, *J* = 8.7 Hz, 1H), 7.54 (d, *J* = 8.5 Hz, 1H), 6.85 (dd, *J* = 8.5, 1.7 Hz, 1H), 6.73 (dd, *J* = 8.7, 0.8 Hz, 1H), 6.60 (d, *J* = 10.0 Hz, 1H), 5.73 (d, *J* = 10.0 Hz, 1H), 4.32 (t, *J* = 7.4 Hz, 2H), 3.91 (s, 3H), 2.93 – 2.78 (m, 2H), 2.53 (s, 3H), 2.25 (s, 3H), 1.95-1.89 (m, 2H), 1.88-1.82 (m, 2H), 1.80-1.74 (m, 2H), 1.46 (s, 6H), 1.42-1.26 (m, 3H); HRMS (FAB) calc for $C_{29}H_{37}N_4O_3$ m/z $[M+H]^+$ 489.2866, found 489.2861; HPLC t_R = 10.636 min, purity 99.0%.

Supplementary information 2

The HSP90 inhibitor HVH-2930 exhibits potent efficacy against trastuzumab-resistant HER2-positive breast cancer

Minsu Park[1,2,*], Eunsun Jung[1,3,*], Jung Min Park[1,2], Soeun Park[1,2], Dongmi Ko[1,2], Juyeon Seo[1,2], Seongjae Kim[1,2], Kee Dal Nam[1,3], Yong Koo Kang[1,3], Lee Farrand[4], Van-Hai Hoang[5], Cong-Truong Nguyen[6], Minh Thanh La[7], Gibeom Nam[8], Hyun-Ju Park[8], Jihyae Ann[7,†], Jeewoo Lee[7,†], Yoon-Jae Kim[1,2,3,†], Ji Young Kim[1,3,†] and Jae Hong Seo[1,2,3,†]

Supplementary Figures and Legends (Figure S1-S19)

Supplementary Figure S1

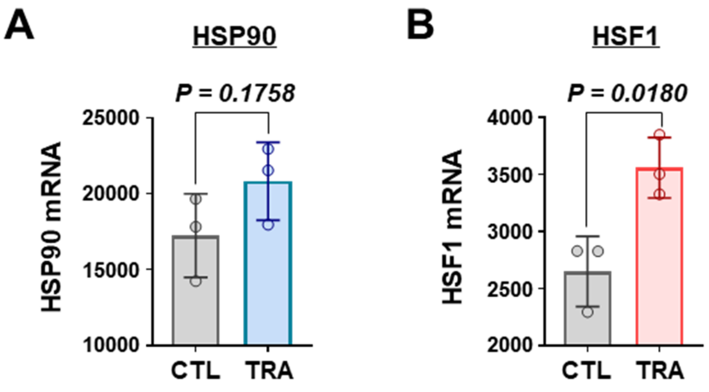

Supplementary Figure S1. Differences in HSP90 and HSF1 expression by trastuzumab responsiveness in HER2-positive breast cancer. (**A-B**) Public GEO dataset analysis for mRNA expression for HSP90 (**A**) and HSF1 (**B**) using gene expression profiles in a patient-derived xenograft (PDX) model, derived from HER2-positive breast cancer with trastuzumab resistance, in the presence or absence of trastuzumab (6 mg/kg, once a week for 3 weeks). [CTL, control vehicle; TRA, trastuzumab].

Supplementary Figure S2

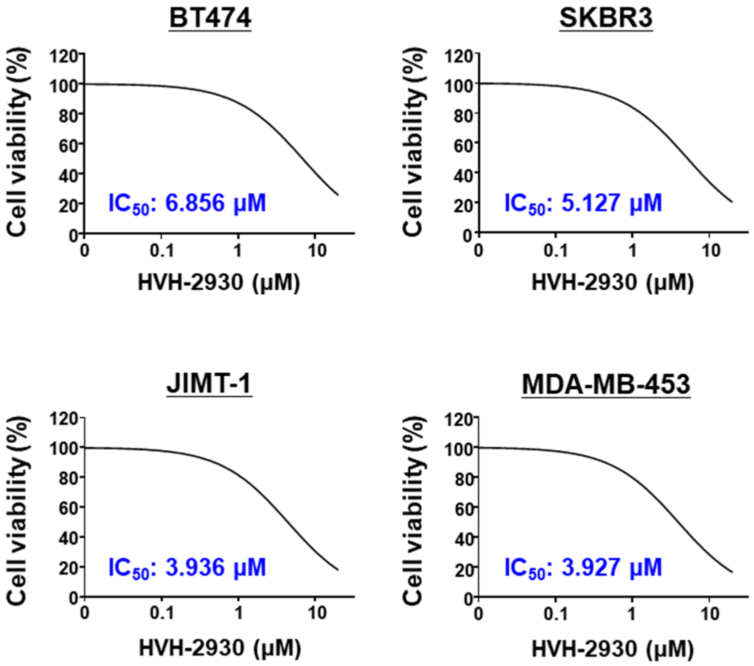

Supplementary Figure S2. Effect of HVH-2930 on cell viability in HER2-positive breast cancer cells, corresponding to Figure 1K in the main text. Trastuzumab-sensitive BT474 and SKBR3 and -resistant JIMT-1 and MDA-MB-453 cells were treated with HVH-2930 (0.1-20 μM) or control vehicle for 72 h. Cell viability and IC_{50} values were determined by MTS assay.

Supplementary Figure S3

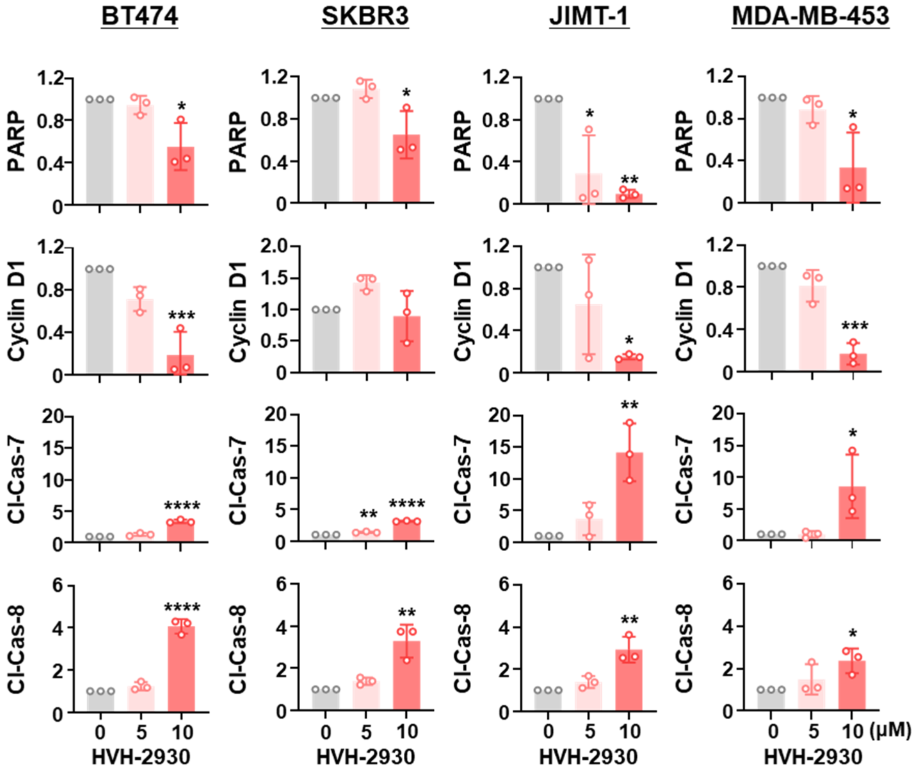

Supplementary Figure S3. Impact of HVH-2930 on the expression of apoptosis-related proteins, corresponding to Figures 2A and 2B in the main text. BT474, SKBR3, JIMT-1 and MDA-MB-453 cells were treated with HVH-2930 (5-10 μM) for 72 h, and immunoblot analysis was performed. Quantitative graphs represent the ratio of PARP, cyclin D1, cleaved caspase-7 and cleaved caspase-8 expression relative to GAPDH in the presence or absence of HVH-2930 (*$p < 0.05$, n = 3). The results are presented as mean ± SEM of at least three independent experiments analyzed by one-way ANOVA followed by Bonferroni's post hoc test.

Supplementary Figure S4

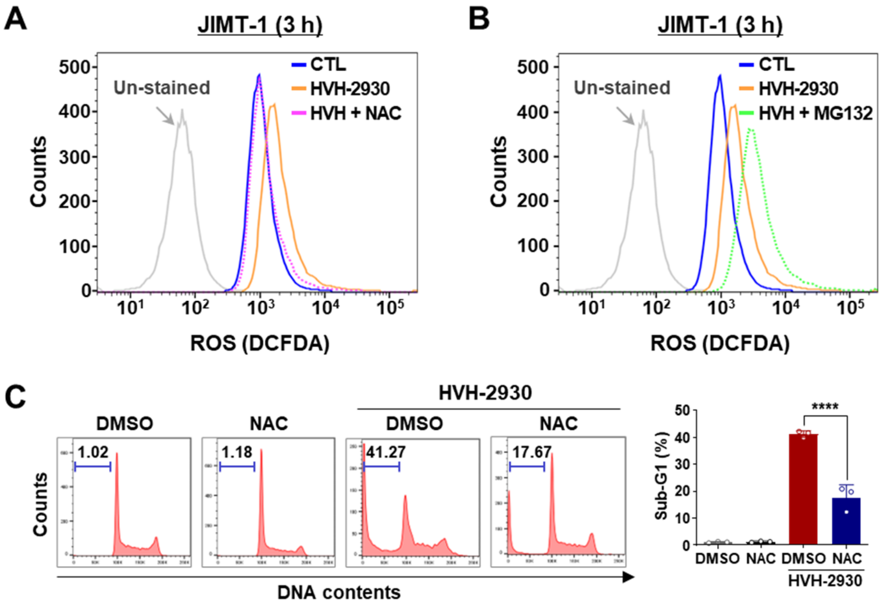

Supplementary Figure S4. ROS scavenger suppresses HVH-2930-induced ROS generation and apoptosis. (**A-B**) JIMT-1 cells were pre-cultured with ROS scavenger NAC (3 mM, 1 h, **A**) or ROS trigger MG132 (10 µM, 1 h, **B**) before HVH-2930 treatment (10 µM, 3 h). The intracellular ROS generation was determined by DCFDA staining. (**C**) Sub-G1 populations were assessed by flow cytometry following exposure to HVH-2930 (10 µM, 72 h) in the presence or absence of NAC (3 mM, 1 h, pretreatment) in JIMT-1 cells. Flow cytometry data were analyzed using FlowJo v10.1 software (****$p < 0.0001$, HVH-2930 treatment alone vs combination treatment with NAC and HVH-2930).

Supplementary Figure S5

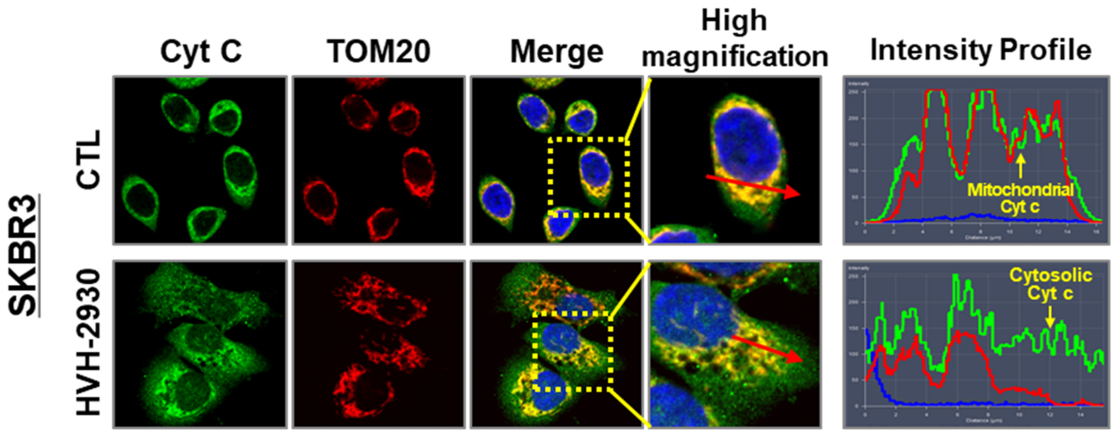

Supplementary Figure S5. Immunofluorescence analysis of cytochrome C (green) and Tom20 (red, mitochondria) with DAPI (blue, nucleus) in SKBR3 cells after treatment with HVH-2930 (10 µM, 24 h). The signal intensity of cytochrome c (green line) and cellular localization (yellow arrows) were analyzed with confocal microscopy using the intensity profiling tool.

Supplementary Figure S6

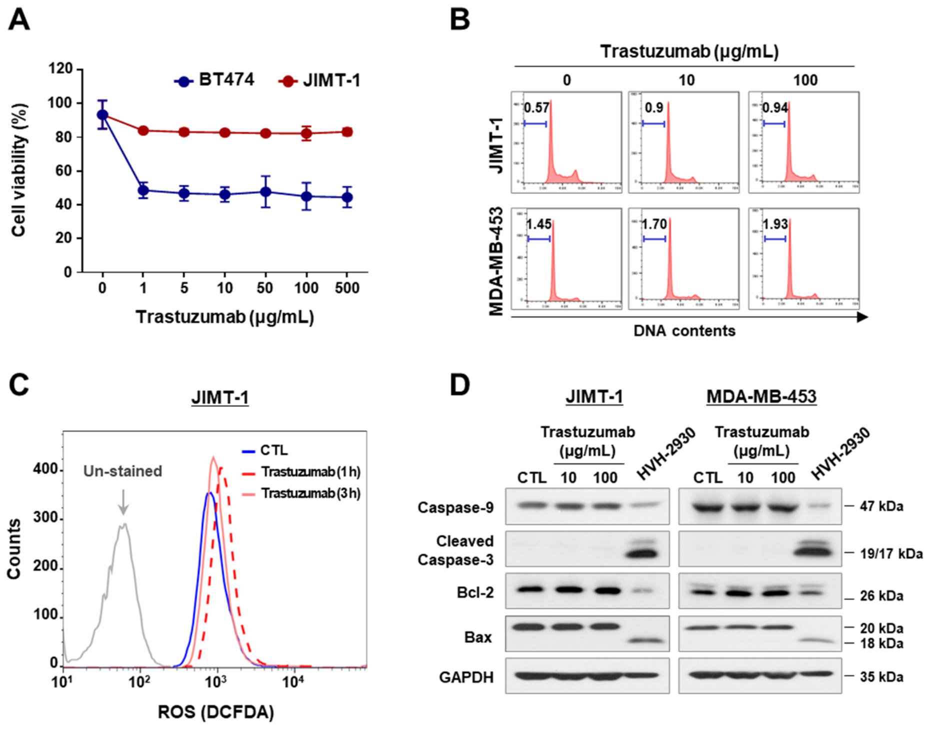

Supplementary Figure S6. Effect of trastuzumab on cell viability and apoptosis in trastuzumab-resistant HER2-positive breast cancer cells. (**A**) BT474 and JIMT-1 cells were treated with trastuzumab (1-500 μg/mL) or a control vehicle for 72 h, and cell viability was determined by MTS assay. (**B**) Sub-G1 populations in JIMT-1 and MDA-MB-453 cells following exposure to trastuzumab (10 and 100 μg/mL, 72 h). (**C**) Measurement of intracellular ROS generations in JIMT-1 cells treated with trastuzumab (100 μg/mL, 1-3 h). (**D**) Changes in the expression of caspase-9, cleaved caspase-3, Bcl-2 and Bax in JIMT-1 and MDA-MB-453 cells after treatment with trastuzumab (10 and 100 μg/mL) or HVH-2930 (10 μM) for 72 h.

Supplementary Figure S7

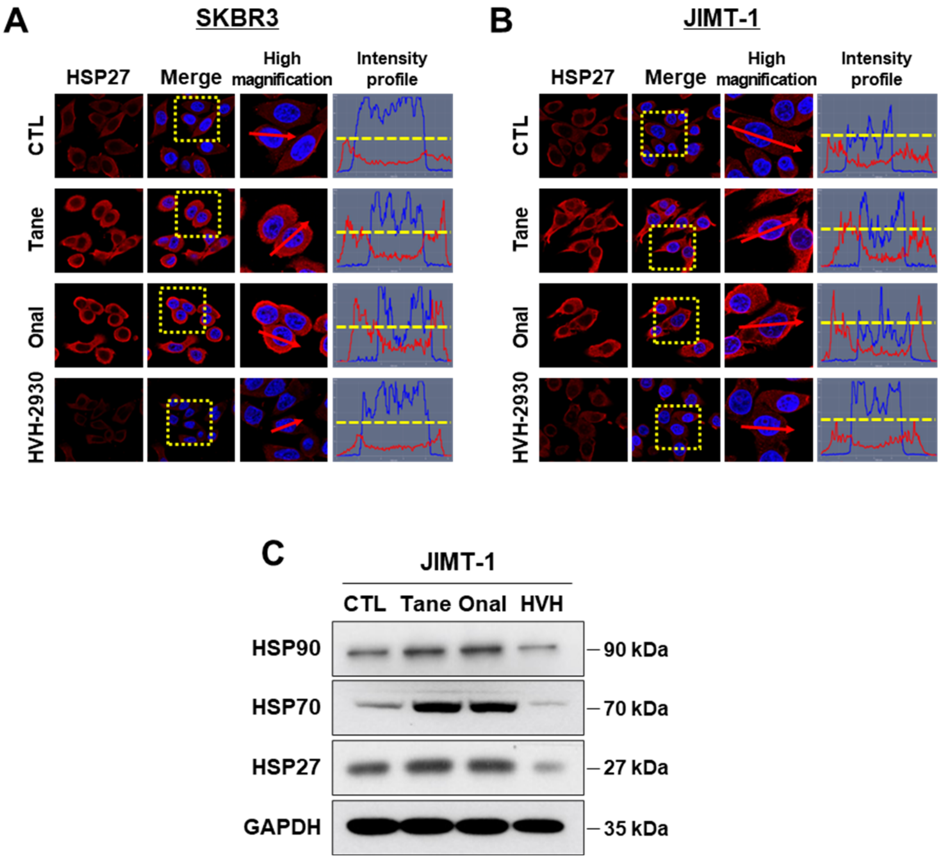

Supplementary Figure S7. Comparison of the effects of HVH-2930 on HSP27 expression. SKBR3 (**A**) and JIMT-1 cells (**B**) were immunostained for HSP27 (red) with DAPI (blue, nuclei), following exposure to HVH-2930, tanespimycin or onalespib (300 nM, 24 h). The fluorescence intensity of these proteins is represented in arbitrary units as defined by the software (yellow dotted line). (**C**) Immunoblot analyses of HSP90, HSP70, HSP27 protein expression in JIMT-1 cells after treatment with tanespimycin (100 nM), onalespib (500 nM), or HVH-2930 (5 µM) for 72 h. GAPDH was used as an internal control. [CTL, control; Tane, tanespimycin; Onal, onalespib; HVH, HVH-2930].

Supplementary Figure S8

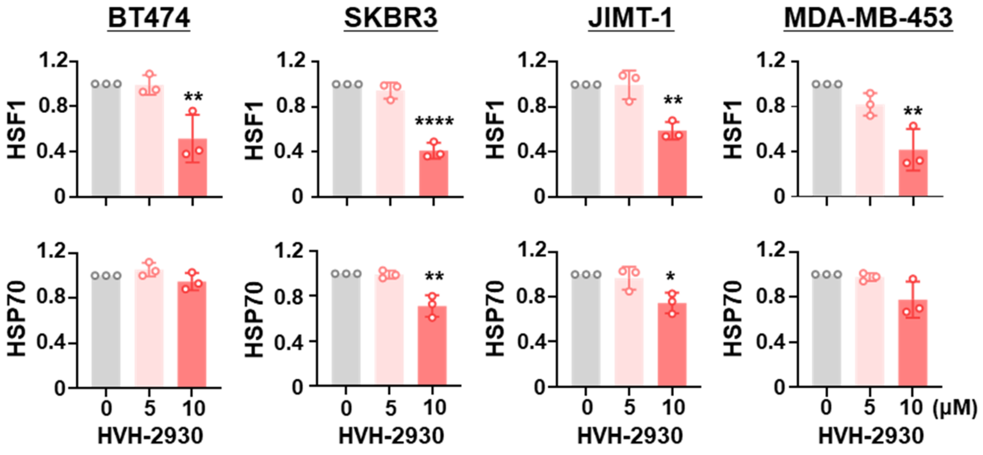

Supplementary Figure S8. Effect of HVH-2930 on the expression of heat shock proteins, corresponding to Figure 3K and 3L in the main text. BT474, SKBR3, JIMT-1 and MDA-MB-453 cells were treated with HVH-2930 (5-10 μM) for 72 h, and immunoblot analysis was performed. Quantitative graphs represent the ratio of HSF1/GAPDH and HSP70/GAPDH in the presence or absence of HVH-2930 (*p < 0.05, n = 3).

Supplementary Figure S9

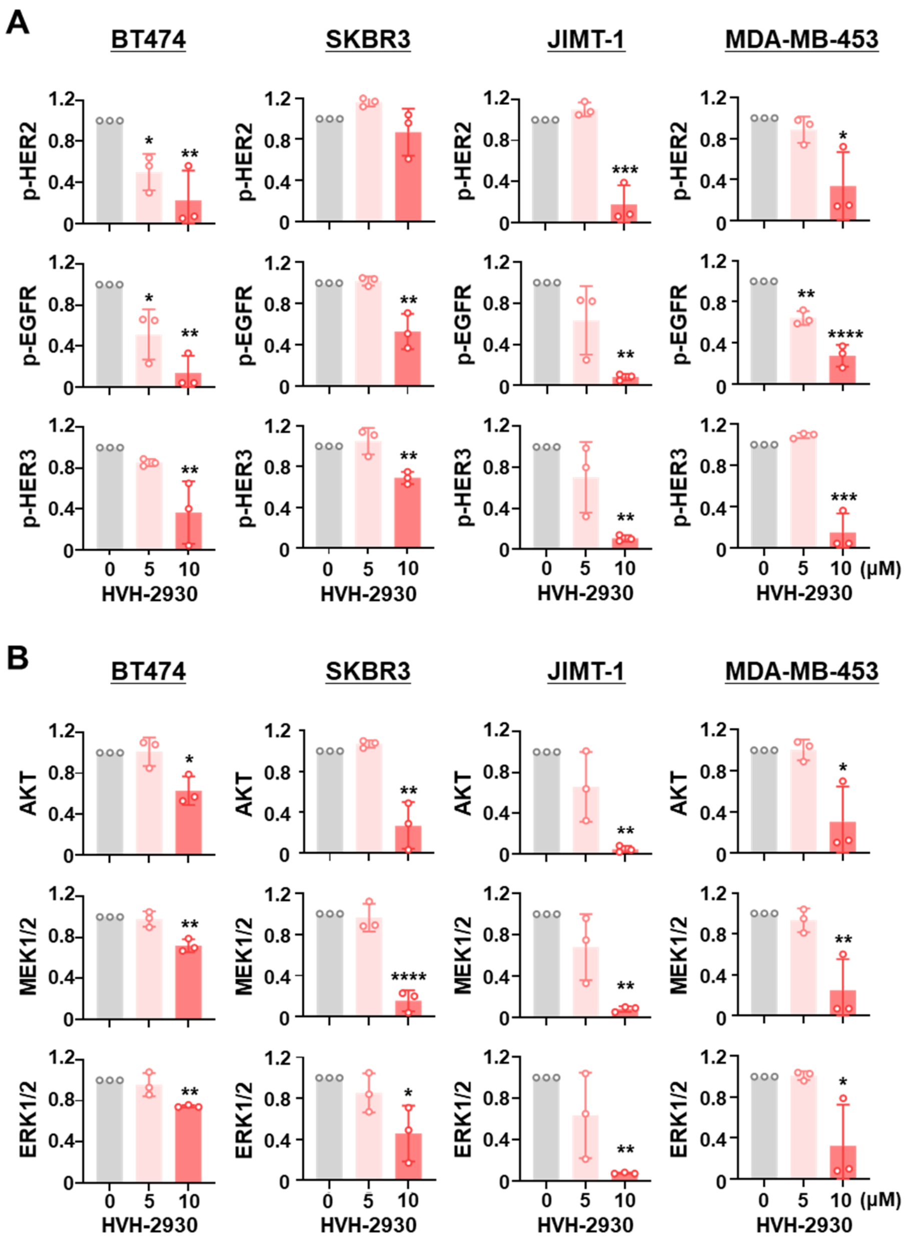

Supplementary Figure S9. HVH-2930 downregulates the expression of HSP90 client proteins, corresponding to Figure 4A-4D in the main text. (**A**) Immunoblot analyses of phospho-HER2 (Y1221/1222), phospho-EGFR (Y1068), phospho-HER3 (Y1289) protein expression in BT474, SKBR3, JIMT-1 and MDA-MB-453 cells following exposure to HVH-2930 (5-10 μM, 72 h). GAPDH was used as a loading control. Quantitative graphs represent the ratio of protein expression relative to GAPDH in the presence or absence of HVH-2930 (*$p < 0.05$, n = 3). (**B**) Immunoblot analyses of AKT, MEK, and ERK protein expression. Quantitative graphs represent the ratio of protein contents (*$p < 0.05$, n = 3).

Supplementary Figure S10

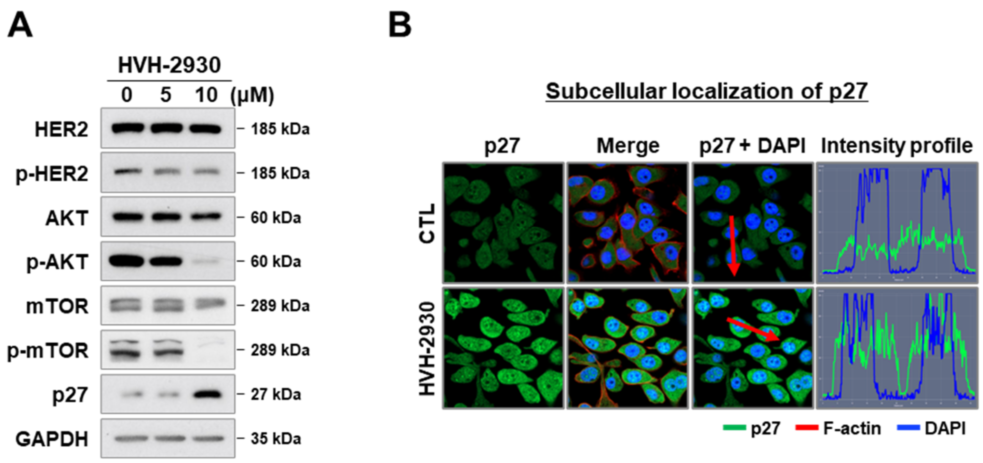

Supplementary Figure S10. Effects of HVH-2930 on the HER2 signaling pathway in trastuzumab-resistant cells. (**A**) Immunoblot analyses of HER2, phospho-HER2 (Y1221/1222), AKT, phospho-AKT (S473), mTOR, phospho-mTOR (S2448), and p27 expression in JIMT-1 cells following exposure to HVH-2930 (5-10 μM, 72 h). GAPDH was used as a loading control. (**B**) JIMT-1 cells were treated with HVH-2930 at 10 μM for 24 h, and immunostained for p27 (green) and F-actin (red, cytoplasm) with DAPI (blue, nucleus). Signal intensity of p27 (green line) and subcellular localization (red arrows) were determined by confocal microscopy using the intensity profile tool.

Supplementary Figure S11

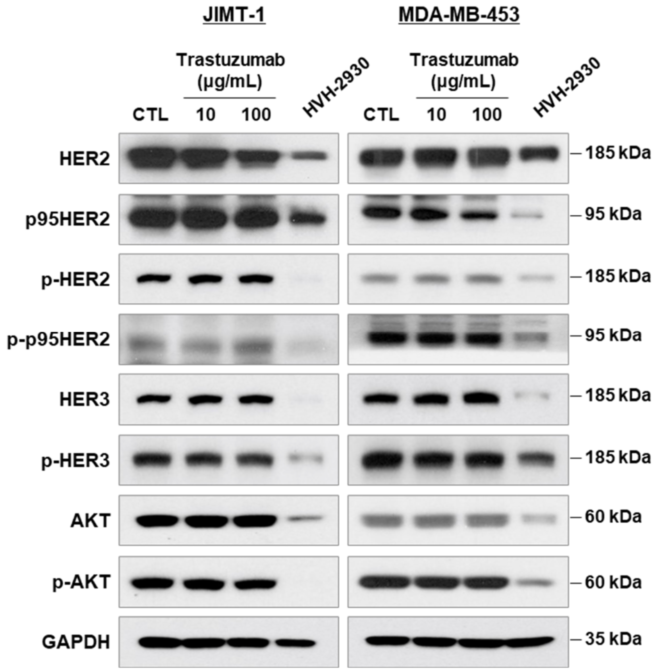

Supplementary Figure S11. Influences of trastuzumab on the HER2 signaling pathway in trastuzumab-resistant cells. Immunoblot analyses of HER2, phospho-HER2 (Y1221/1222), p95HER2, phospho-p95HER2, HER3, phospho-HER3 (Y1289), AKT, phospho-AKT (S473) protein expression in JIMT-1 and MDA-MB-453 cells following exposure to trastuzumab (10 and 100 µg/mL) or HVH-2930 (10 µM) for 72 h.

Supplementary Figure S12

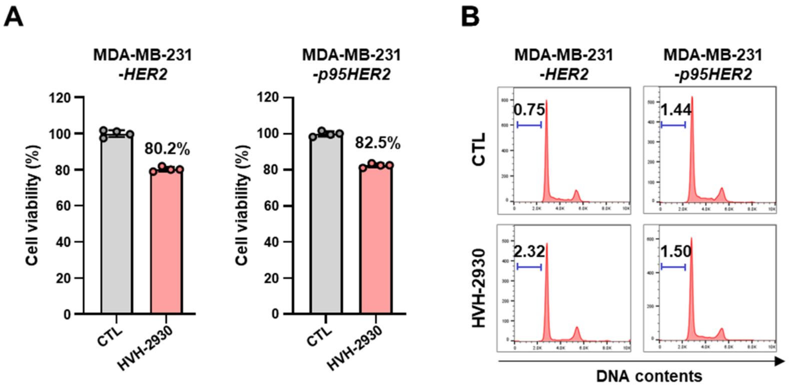

Supplementary Figure S12. Influence of HVH-2930 on cell viability and apoptosis used to establish an experimental metastasis model. HER2- and p95HER2-overexpressing MDA-MB-231 cells were treated with HVH-2930 (10 µM) or a control vehicle for 24 h. (**A**) Cell viability was determined by MTS assay, and (**B**) Sub-G1 populations were assessed by flow cytometry.

Supplementary Figure S13

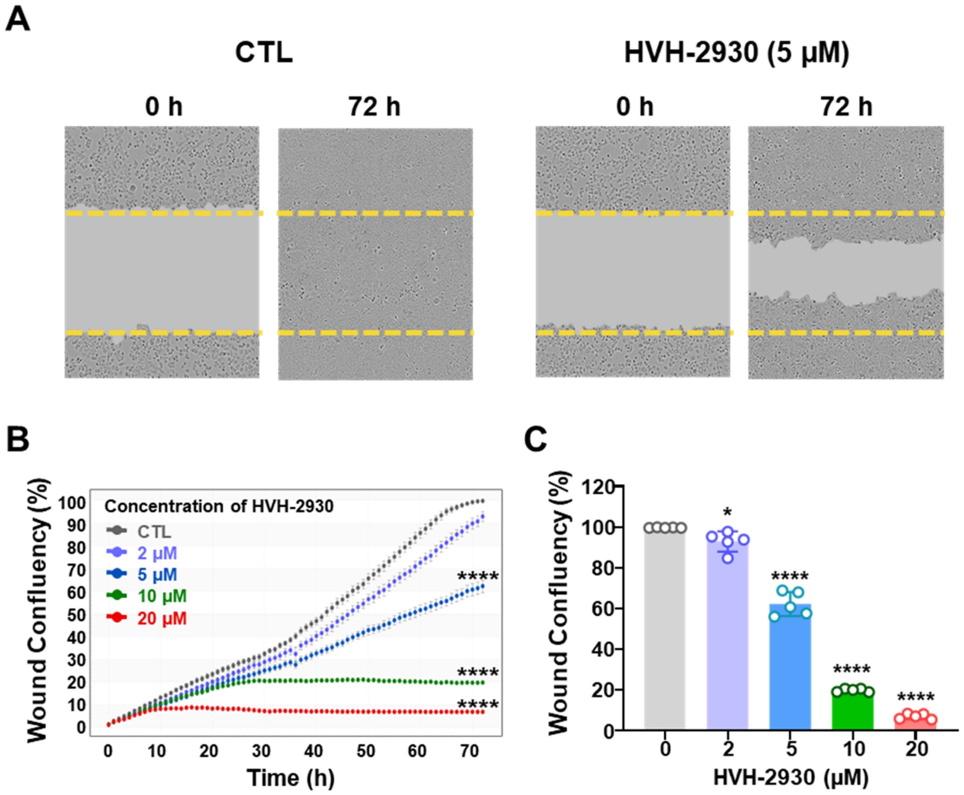

Supplementary Figure S13. Influence of HVH-2930 on cell migration in JIMT-1 cells. (**A-C**) JIMT-1 cells were treated with HVH-2930 (1-20 μM) for 72 h. (**A**) Representative images of wound closure by cell migration at 0 and 72 h after HVH-2930 treatment (5 μM). The yellow dotted line indicates the edge of the scratched wound. (**B**) The kinetic analysis of cell migration was determined using the IncuCyte™ Live-Cell Imaging System and quantified for the indicated time duration (****$p < 0.0001$, n = 6). (**C**) The quantitative graph represents the relative wound density (%) in JIMT-1 cells at 72 h (*$p < 0.05$).

Supplementary Figure S14

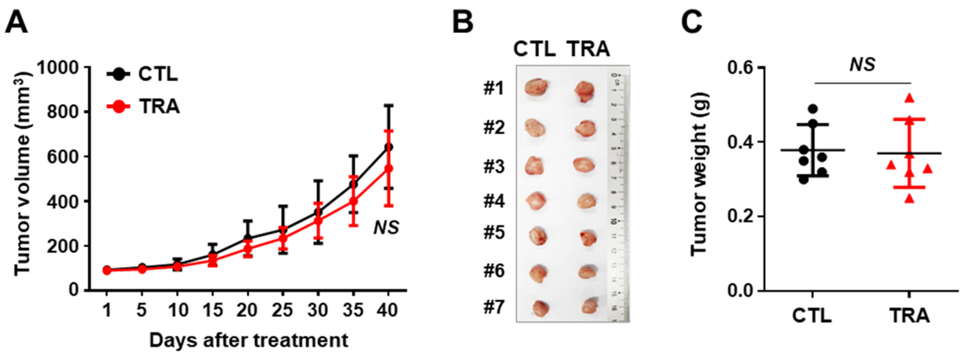

Supplementary Figure S14. Effect of trastuzumab on tumor growth in trastuzumab-resistant JIMT-1 xenografts. (**A-C**) JIMT-1 cells (3×10^6) were injected into the mammary fat pads of BALB/c nude mice (n = 7/each group). Following exposure to trastuzumab (initial loading dose of 4 mg/kg followed by 2 mg/kg every week) for 40 days, tumor growth (**A**, NS, not significant), tumor burden (**B**) and tumor weight (**C**, NS) were evaluated. [CTL, control; TRA, trastuzumab]

Supplementary Figure S15

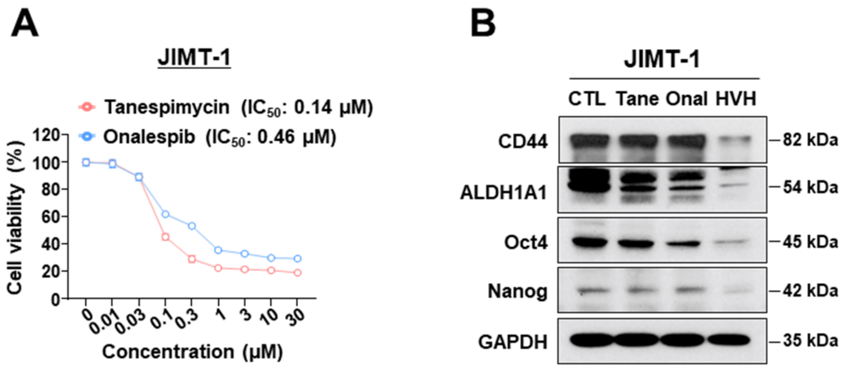

Supplementary Figure S15. Effects of N-terminal HSP90 inhibitors tanespimycin and onalespib on CSC-like properties. (**A**) JIMT-1 cells were treated with onalespib and tanespimycin (0.01-30 μM) for 72 h, and cell viability and IC_{50} values were determined by MTS assay. (**B**) Immunoblot analyses for CD44, ALDH1A1, Oct4 and Nanog in JIMT-1 cells after treatment with tanespimycin (100 nM), onalespib (500 nM) or HVH-2930 (5 μM) for 72 h.

Supplementary Figure S16

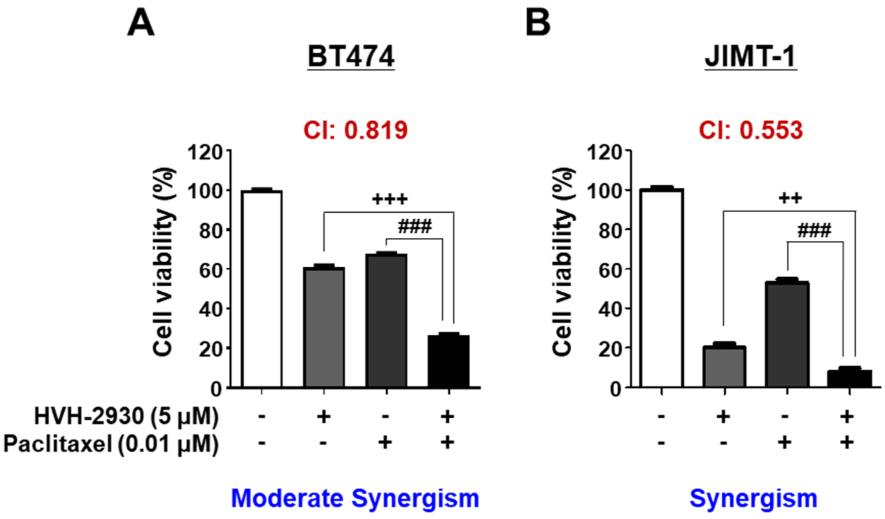

Supplementary Figure S16. The combination effects of HVH-2930 and paclitaxel on cell viability in HER2-positive breast cancer cells, corresponding to Figure 7A in the main text. BT474 (**A**) and JIMT-1 cells (**B**) were treated at the indicated concentrations of HVH-2930 (0-5 μM) and paclitaxel (0-0.1 μM) for 72 h, and cell viability was assessed by MTS assay (##$p < 0.01$, HVH-2930 only vs combination; +++$p < 0.001$, PTX only vs combination).

Supplementary Figure S17

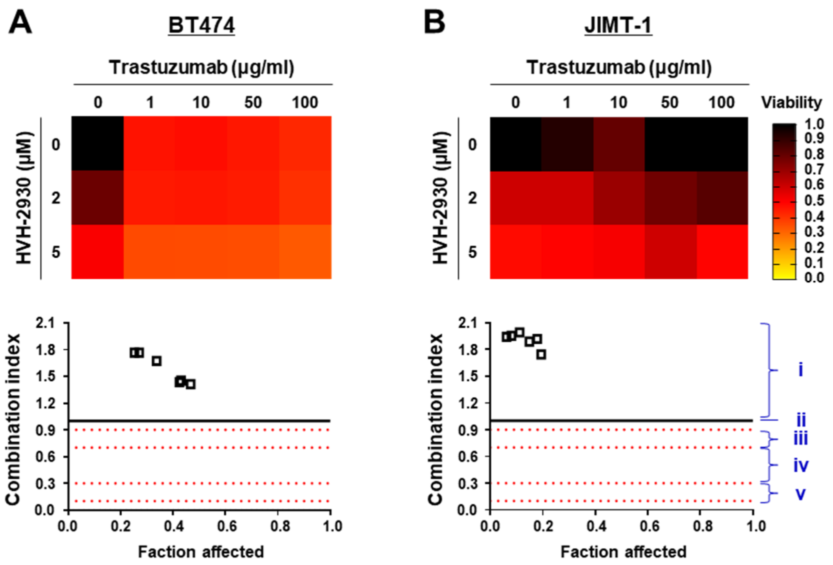

Supplementary Figure S17. The combined effect of HVH-2930 and trastuzumab on the viability of HER2-positive breast cancer cells. BT474 (**A**) and JIMT-1 cells (**B**) were treated at the indicated concentrations of HVH-2930 (0-5 µM) and trastuzumab (0-100 µg/mL) for 72 h. Cell viability was analyzed by MTS assay. Color intensity represents relative cell viability compared with DMSO control. The bottom panel shows combination indices for HVH-2930 and trastuzumab in each cell line. The combination index (CI) was used to quantify synergism or antagonism for two drugs, where CI < 1, = 1, and > 1 indicate synergism, independence, and antagonism, respectively. Fraction affected (Fa) of a group was calculated as Fa = percent inhibition of cell viability/100. [i, antagonism; ii, addictive effect; iii, moderate synergism; iv, synergism; v, strong synergism]

Supplementary Figure S18

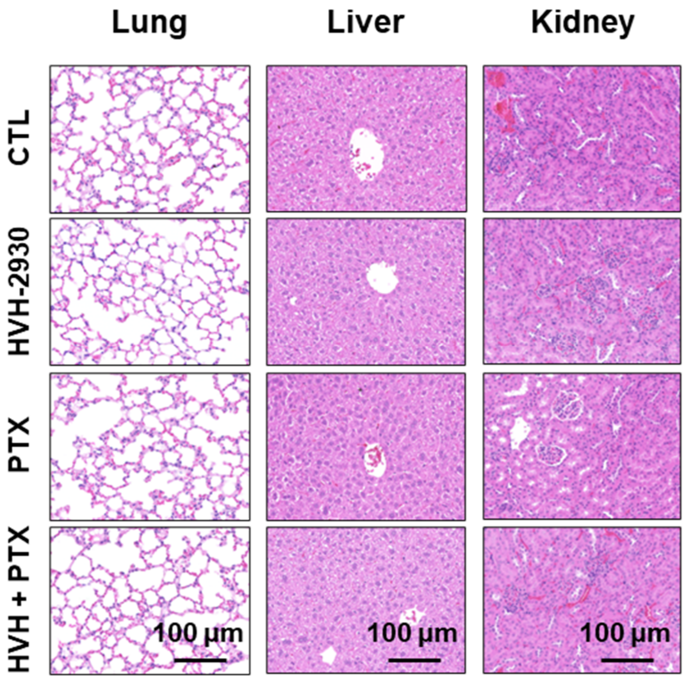

Supplementary Figure S18. Representative histological images of lung, liver and kidney sections were obtained using hematoxylin and eosin (H&E) staining. Histochemical microphotographs were taken using a Carl Zeiss Axio Scan.Z1 and shown at high magnification (scale bar: 100 μm).

Supplementary Figure S19

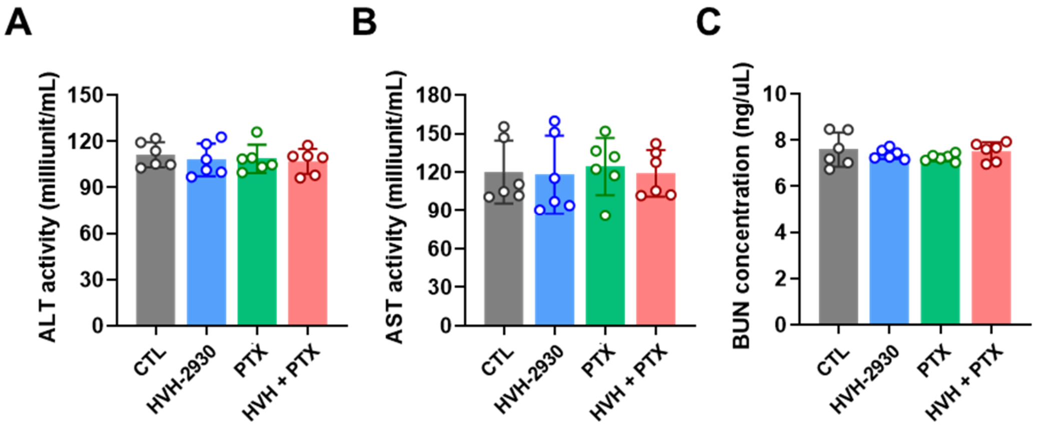

Supplementary Figure S19. Effects of HVH-2930 in combination with paclitaxel on serum biochemical parameters of liver and kidney function. Blood biochemical analysis indicated there was no significant change in serum ALT (**A**, NS, not significant), AST (**B**, NS) or BUN (**C**, NS)
